# F13A1‐Mediated Macrophage Activation Promotes MASH Progression via the PKM2/HIF1A Pathway

**DOI:** 10.1002/advs.202518128

**Published:** 2025-12-19

**Authors:** Qianrang Lu, Meiching Ong, Xuewen Yi, Muqiong Xing, Xinyao Tian, Ke Zhang, Ling Lu, Hao Wang, Xiaohan Lin, Jun Fang, Aibo Mu, Jiaying Cao, Jingyu Jiang, Feng Gao, Hongjun Li, Baohong Wang, Qi Ling

**Affiliations:** ^1^ Department of Hepatobiliary and Pancreatic Surgery The First Affiliated Hospital Zhejiang University School of Medicine Hangzhou China; ^2^ NHC Key Laboratory of Combined Multi‐Organ Transplantation Hangzhou China; ^3^ State Key Laboratory of Advanced Drug Delivery and Release Systems College of Pharmaceutical Sciences Zhejiang University Hangzhou China; ^4^ Department of Hepatobiliary and Pancreatic Surgery The Second Affiliated Hospital Zhejiang University School of Medicine Hangzhou China; ^5^ Jiangsu Key Laboratory of Organ Transplantation and Transplant Immunology Research Unit of Liver Transplantation and Transplant Immunology Chinese Academy of Medical Sciences Hepatobiliary Center The Affiliated Hospital of Xuzhou Medical University Xuzhou China; ^6^ Collaborative Innovation Center for Cancer Personalized Medicine Nanjing Medical University Nanjing China; ^7^ Hepatobiliary Center The First Affiliated Hospital Nanjing Medical University Research Unit of Liver Transplantation and Transplant Immunology Chinese Academy of Medical Sciences Nanjing Jiangsu China; ^8^ Key Laboratory of Advanced Drug Delivery Systems of Zhejiang Province College of Pharmaceutical Sciences Zhejiang University Hangzhou China; ^9^ Liangzhu Laboratory Zhejiang University Hangzhou China; ^10^ State Key Laboratory for Diagnosis and Treatment of Infectious Diseases National Clinical Research Center for Infectious Diseases Collaborative Innovation Center for Diagnosis and Treatment of Infectious Diseases The First Affiliated Hospital Zhejiang University School of Medicine Hangzhou China

**Keywords:** factor XIII, HIF1A, PKM2, macrophage activation, MASH

## Abstract

Macrophages are central mediators of hepatic inflammation and fibrosis in metabolic‐associated steatohepatitis (MASH), yet the mechanisms driving their activation remain unclear. Integration of four human single‐nucleus transcriptomic datasets identified Coagulation Factor XIII‐A (F13A1)‐positive macrophages as the predominant subset in MASH livers, a finding validated in patient samples and murine models. Lipid‐stressed hepatocytes induce F13A1 expression through a sphingosine‐1‐phosphate (S1P)‐dependent mechanism. Silencing F13A1 suppressed the pro‐inflammatory phenotype and alleviated hepatic injury in vivo. Mechanistically, F13A1 directly interacted with pyruvate kinase M2 (PKM2), promoting its dimerization, a process enhanced by intracellular calcium levels. Dimerized PKM2 translocated into the nucleus and upregulates interleukin‐1 beta (IL1B) expression via the PKM2/HIF1A (Hypoxia‐inducible factor 1‐alpha) axis. In addition, F13A1 enhanced the Warburg effect in macrophages through PKM2‐mediated metabolic reprogramming. Pharmacologic activation of PKM2 with DASA‐58 abrogated F13A1‐driven inflammation, and PEG‐PLA micelle‐mediated delivery of DASA‐58 ameliorated hepatic inflammation in vivo. These findings establish F13A1 as a critical driver of macrophage‐mediated inflammation in MASH and highlight the F13A1/PKM2/HIF1A pathway as a promising therapeutic target.

## Graphical abstract

In fatty liver disease, hepatocytes exposed to palmitate release S1P, which activates calcium signaling in macrophages. Elevated calcium enhances the activity of F13A1, driving PKM2 dimerization. The PKM2 dimers cause Warburg effect, translocate to the nucleus, cooperate with HIF1A, and upregulate IL1B expression, ultimately promoting classical activation of macrophages and contributing to hepatic inflammation.

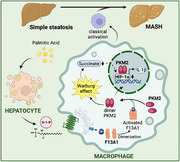

## 1 Introduction

Metabolic dysfunction‐associated steatotic liver disease (MASLD), the most common chronic liver disease worldwide, encompasses a spectrum ranging from benign steatosis to metabolic dysfunction‐associated steatohepatitis (MASH), fibrosis, and eventually cirrhosis. During the transition to MASH, hepatic inflammation plays a central pathogenic role by driving hepatocyte injury and fibrotic remodeling [1]. Among immune populations, hepatic macrophages, comprising resident Kupffer cells and monocyte‐derived macrophages, are key mediators of inflammation and tissue damage. Recent single‐cell and spatial transcriptomic studies have revealed that macrophages in MASLD adopt a pro‐inflammatory phenotype characterized by metabolic reprogramming; secretion of chemokines such as CCL2 and CXCL10; production of cytokines including tumor necrosis factor‐α (TNF‐α) and interleukin‐1β (IL‐1β); and increased expression of the inflammasome sensor NLRP3, which is essential for IL‐1β maturation [2, 3, 4]. These macrophages are activated by diverse hepatic and extrahepatic cues, including lipotoxic lipids, hepatocyte‐derived damage‐associated molecular patterns (DAMPs), gut‐derived endotoxins, and local inflammatory cytokines [5, 6]. Despite these advances, the intracellular regulators and molecular programs that sustain macrophage activation in the steatotic liver remain poorly defined.

F13A1, traditionally recognized as a coagulation factor that stabilizes fibrin clots through crosslinking, also exhibits functions beyond hemostasis. It has been implicated in obesity [7, 8], liver fibrosis [9], and atherosclerosis [10], yet its role in MASH remains largely unexplored. Recent studies using lineage‐specific knockout models have shown that F13A1 is expressed primarily in platelet lineages and myeloid cells, with the latter representing the major source of circulating factor XIII‐A [11, 12]. To date, research on F13A1 in macrophages has predominantly focused on its secreted, extracellular roles in matrix crosslinking and wound healing [13, 14, 15]. However, F13A1 also exerts important intracellular functions as a calcium‐dependent transglutaminase [16, 17]. Comprehensive investigations into these intracellular activities of F13A1 in macrophages, particularly in inflammatory and metabolic contexts such as MASH, remain scarce.

PKM2, an alternatively spliced isoform of the pyruvate kinase muscle (PKM) gene, is mainly expressed in cancer cells [18], proliferating cells [19], and immune cells like macrophages [20] and T cells [21]. PKM2 exists either as an active tetramer that catalyzes glycolytic flux or as a dimer capable of nuclear translocation, which also induces the Warburg effect [22]. In macrophages, lipopolysaccharide (LPS) is the most extensively studied stimulus inducing PKM2 dimerization and nuclear import, where nuclear PKM2 acts as a coactivator of hypoxia‐inducible factor 1‐alpha (HIF1A) to promote transcription of IL1B and other pro‐inflammatory genes [20, 23, 24]. Although the PKM2/HIF1A axis has been recognized as a central link between metabolism and inflammation, the upstream mechanisms that govern PKM2 dimerization in macrophages remain poorly defined. This study investigates the intracellular role of F13A1 in macrophage activation under steatotic conditions and elucidates its potential interaction with PKM2‐mediated inflammatory signaling.

## 2 Results

### 2.1 Elevated F13A1+ Macrophages in MASH

**FIGURE 1:**
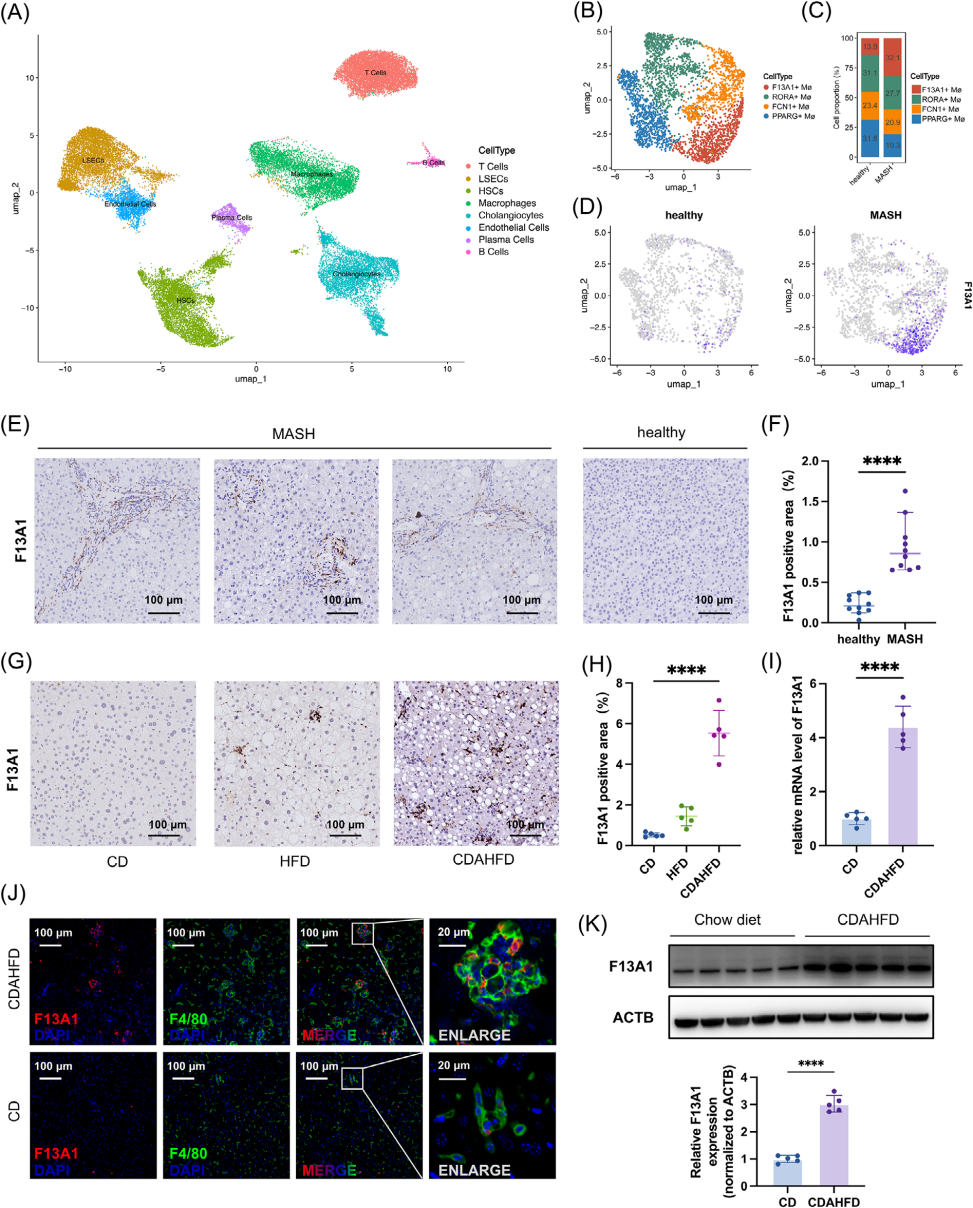
Increased Proportion of F13A1^+^ Macrophages in MASH. (A) Clustering of 33,223 NPCs into 8 clusters from 6 healthy individuals and 7 MASH patients. (B) Subclustering of 3326 macrophages into 4 subpopulations. (C) Proportions of each macrophage subset in normal vs. MASH livers, ranked by abundance in MASH group. (D) UMAP plots showing F13A1 distribution split by disease status. (E,F) Representative IHC images of F13A1 expression in human liver (normal n = 10, MASH n = 10), and quantification of F13A1‐positive area (F). (G,H) F13A1 IHC staining of mouse livers from HFD‐fed mice (12 weeks), CDAHFD‐fed mice (8 weeks), or CD controls (n = 5 per group), and quantification of hepatic F13A1‐positive area (H). (J) Immunofluorescence staining of F4/80 (green), F13A1 (red), and nuclei (DAPI, blue) in CD or CDAHFD‐fed mouse liver. (I,K) Magnetic bead isolation of hepatic macrophages from CDAHFD‐fed mice, followed by qPCR (I) and Western blot, along with relative quantification of the Western blot (K). Data are presented as mean ± standard deviation. For (F, I, K), significance was determined by Student's *t*‐test. For (H), significance was determined by one‐way ANOVA. ns = no significance, *p < 0.05, **p < 0.01, ***p < 0.001, ****p < 0.0001. Abbreviations: NPCs, non‐parenchymal cells; MASH, metabolic dysfunction‐associated steatohepatitis; CD, chow diet; HFD, high‐fat diet; CDAHFD, choline‐deficient, L‐amino acid‐defined, high‐fat diet; IHC, immunohistochemistry.

To investigate the role of macrophages in fatty liver disease, we integrated four human liver snRNA‐seq GEO datasets (GSE212046, GSE174748 [25], GSE185477 [26], GSE189175 [27]; healthy = 6, MASH = 7) (Figure S1A). This analysis identified eight non‐parenchymal cell populations (Figure 1A; Figure S1B). Further clustering of hepatic macrophages identified 4 distinct subsets (Figure 1B; Figure S1C). One subset constituted the largest macrophage population in fatty liver and was markedly expanded in MASH compared with healthy controls (Figure 1C,D). Differential expression analysis revealed F13A1 as the most prominent marker gene defining this subset (Table S1).

To validate these findings, we examined liver tissues from 20 human samples (healthy = 10, MASH = 10). Immunohistochemistry (IHC) showed significantly increased infiltration of F13A1^+^ cells in MASH livers (Figure 1E,F). Consistently, high‐fat diet (HFD, 12 weeks) and CDAHFD (8 weeks) mouse models of steatosis and steatohepatitis displayed elevated F13A1^+^ cells infiltration, with the highest levels observed in CDAHFD‐induced MASH (Figure 1G,H). Transcriptomic analysis of the GSE135251 [28] dataset further demonstrated a strong association between *F13A1* expression and MASLD activity score (OR = 2.53, p = 7.88×10^−8^) (Figure S1D). The GSE167523 [29] dataset similarly showed increased *F13A1* expression during the progression from simple steatosis to steatohepatitis (Figure S1E).

To determine the identity of F13A1⁺ cells, we performed immunofluorescence staining on liver sections from MASH mice, which revealed that these cells were almost exclusively macrophages. Their numbers were markedly increased compared with controls, in which F13A1⁺ cells were nearly absent (Figure 1J). Consistent with this observation, re‐analysis of the previous single‐cell transcriptomic data confirmed that F13A1 expression is highly specific to macrophages (Figure S1G), which was in agreement with earlier reports [11]. Primary hepatic macrophages isolated from MASH mice also exhibited significantly elevated F13A1 levels by qPCR and Western blot (Figure 1I,K). To explore the origin of F13A1^+^ macrophages, we analyzed the GSE138778 [30] dataset and found that monocyte‐derived Kupffer cells in MASH mice expressed higher levels of *F13a1* than embryo‐derived Kupffer cells, with even greater differences when compared to Kupffer cells from healthy controls (Figure S1F). Notably, the presence of F13A1^+^ macrophages was also observed across multiple murine liver injury models, including bile duct ligation, liver ischemia‐reperfusion, and liver transplantation (Figure S1H–J).

Collectively, these data indicate that F13A1^+^ macrophages expanded during MASH progression and were likely derived from infiltrating monocytes, suggesting a potential role for this macrophage subset in disease pathogenesis.

### 2.2 F13A1 Is Induced by Hepatocyte‐Derived Signals and Promotes Pro‐Inflammatory Phenotype in Macrophages

**FIGURE 2:**
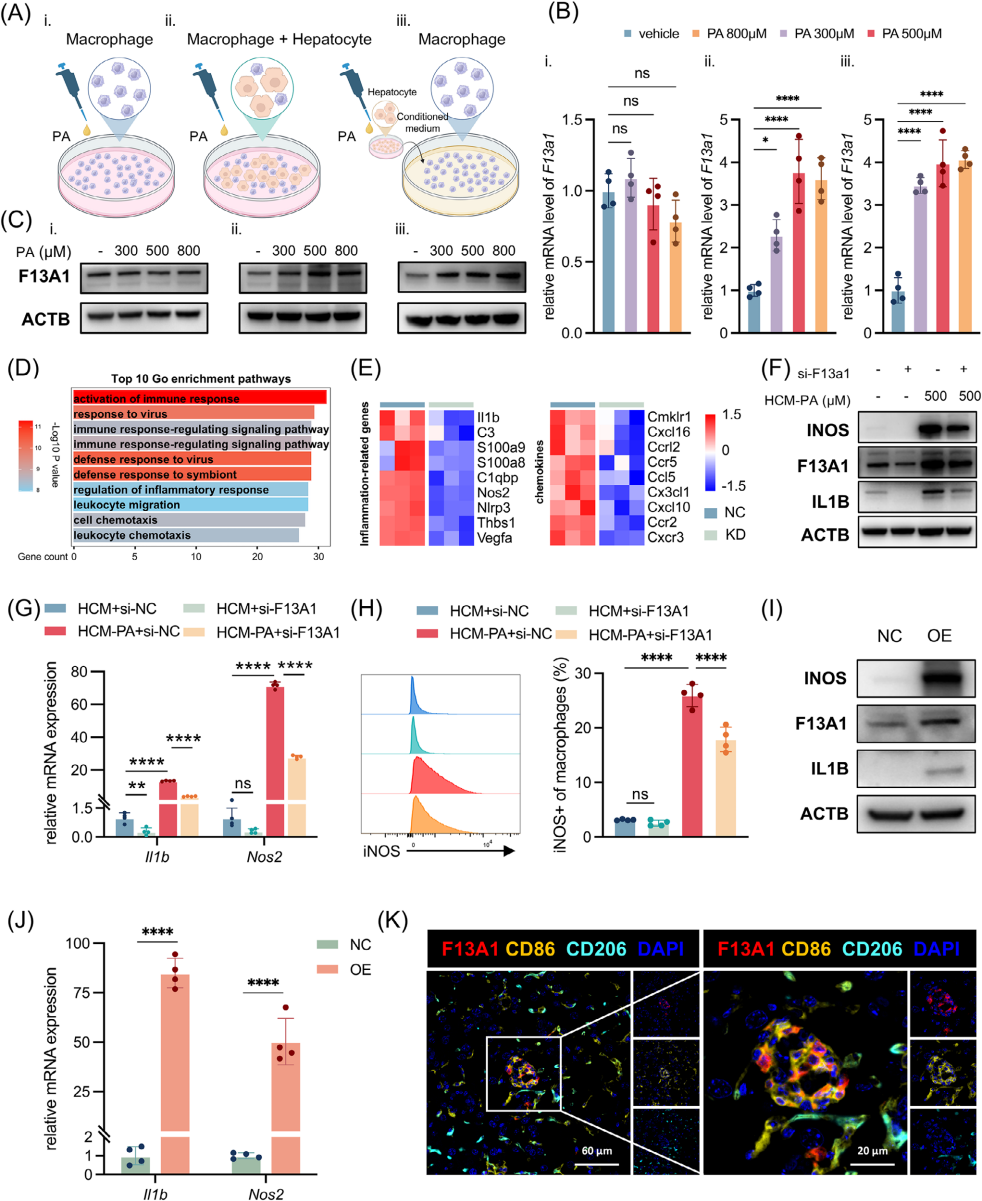
Hepatocyte‐derived signals induce macrophage F13A1 expression, and promotes pro‐inflammatory phenotype in macrophages. (A) Three in vitro approaches simulating a lipotoxic hepatic microenvironment: (i) direct palmitic acid (PA) exposure to macrophages; (ii) hepatocyte–macrophage co‐culture followed by PA; (iii) stimulation of macrophages with supernatant from palmitic acid‐treated hepatocytes. (B,C) Macrophages were stimulated under these three different approaches using 300, 500, or 800 µm palmitic acid. F13A1 expression determined by qPCR (B) and Western blotting (C). (D,E) BMDMs were transfected with F13A1 siRNA or NC siRNA for 24 h. (D) Top 10 enriched GO terms among significantly downregulated genes (*p* < 0.05, log2FoldChange < –0.5), ranked by gene count, and (E) heatmap of downregulated inflammation‐related genes and chemokines (*p* < 0.05). (F–H) BMDMs were transfected with F13A1 siRNA or NC siRNA for 12 h followed by HCM/HCM‐PA induction for additional 24 h; gene expression of F13A1, IL1B, and NOS2 was measured by qPCR (F) and Western blotting (G); proportion of iNOS‐positive macrophages was analyzed via flow cytometry (H). (I,J) RAW246.7 cells were transfected with F13A1 or NC plasmids for 24 h; subsequent analysis of gene expression was *Il1b, Nos2* measured by qPCR (I), and F13A1, IL1B, iNOS were measured by Western blotting (J). (K) Immunofluorescence staining for F13A1 (red), CD86 (gold), CD206 (cyan), and DAPI nuclear counterstain (blue) was performed on liver sections from CDAHFD mice (8 weeks). Data are presented as mean ± standard deviation. For (B) and (H), significance was determined by one‐way ANOVA. For (G), significance was determined by two‐way ANOVA. For (J), significance was determined by Student's *t*‐test. ns = no significance, *p < 0.05, **p < 0.01, ***p < 0.001, ****p < 0.0001. Abbreviations: PA, palmitic acid; HCM, hepatocyte‐conditioned medium; HCM‐PA, palmitic acid–treated hepatocyte‐conditioned medium; BMDMs, bone marrow–derived macrophage; GO, Gene Ontology; NC, negative control; siRNA, small interfering RNA; CDAHFD, choline‐deficient, L‐amino acid‐defined, high‐fat diet.

To mimic a lipotoxic hepatic microenvironment in vitro, bone marrow‐derived macrophages (BMDMs) were stimulated using three approaches: 1) direct exposure to palmitic acid (PA), 2) co‐culture with primary hepatocytes followed by palmitic acid (PA) treatment, and 3) incubation with conditioned medium collected from PA‐treated or vehicle‐treated hepatocytes (HCM‐PA or HCM, respectively) (Figure 2A). Notably, only HCM‐PA or hepatocyte co‐culture induced F13A1 expression in BMDMs, whereas direct PA stimulation alone had no effect (Figure 2B,C; Figure S2A), indicating hepatocyte‐derived mediators, rather than palmitic acid alone, were responsible. Furthermore, HCM‐PA or hepatocyte co‐culture elicited a more robust pro‐inflammatory activation of macrophages than PA alone, as reflected by elevated *Nos2* and *Il1b* expression (Figure S2B).

To investigate the role of F13A1 in macrophage activation, we silenced F13A1 using lipid nanoparticle (LNP)—mediated delivery of F13A1‐specific siRNA, followed by transcriptomic profiling. Genes downregulated upon F13A1 knockdown were enriched in pathways related to immune activation and chemotactic responses (Figure 2D; Figure S2C–E), including canonical pro‐inflammatory mediators such as *Il1b, Nlrp3, Cxcl10*, and *Nos2* (Figure 2E). These findings suggested that F13A1 contributes to the pro‐inflammatory polarization of macrophages. To further evaluate this in a fatty liver milieu, BMDMs were exposed to HCM or HCM‐PA. Expression of IL1B and inducible nitric oxide synthase (iNOS) was markedly elevated after HCM‐PA stimulation, and this effect was significantly attenuated by F13A1 knockdown (Figure 2F,G; Figure S2F). Similar reductions were observed for *Cxcl10* and *Nlrp3* (Figure S2G). Flow cytometry further confirmed that F13A1 knockdown markedly reduced the proportion of iNOS^+^ macrophages induced by HCM‐PA (Figure 2H). Conversely, overexpression of F13A1 plasmid in RAW264.7 cells increased IL1B and iNOS expression (Figure 2I,J; Figure S2H), along with elevated *Cxcl10* and *Nlrp3* levels (Figure S2I).

Finally, multiplex immunofluorescence staining of MASH liver tissue revealed that F13A1 exhibited stronger colocalization with the pro‐inflammatory macrophage marker CD86 than with the anti‐inflammatory marker CD206 (Figure 2K), further supporting the pro‐inflammatory nature of F13A1^+^ macrophages in MASH.

Overall, these findings indicate that increased F13A1 gene expression promotes classical activation of macrophages, which is enhanced by hepatocyte‐derived signals under lipid overload.

### 2.3 Targeting F13A1 in Macrophages Alleviates Liver Inflammation in MASH

**FIGURE 3:**
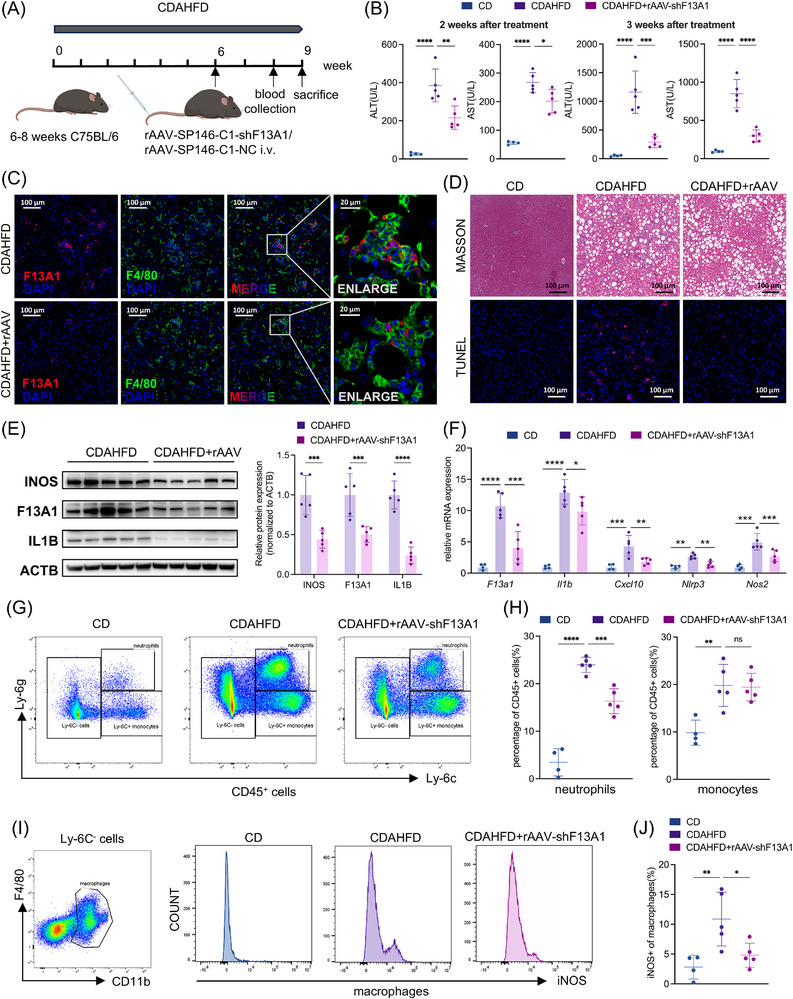
rAAV‐mediated F13A1 silencing in hepatic macrophages ameliorates liver inflammation in MASH mice. (A) CDAHFD‐fed mice were injected with rAAV9‐sp146‐C1‐shF13A1 or rAAV9‐sp146‐C1‐NC (2×10^11 vg/mouse) at week 6; blood was collected after 2 weeks; mice were sacrificed, and sample were collected after 3 weeks (n = 4–5/group). (B) Serum ALT and AST at weeks 2 and 3 post‐treatment. (C) Immunofluorescence staining of F4/80 (green), F13A1 (red), and nuclei (DAPI, blue) was performed in CDAHFD‐fed mouse treated with rAAV9‐sp146‐C1‐NC or rAAV9‐sp146‐C1‐shF13A1. (D) Masson and TUNEL staining of mouse liver. (E) Western blot of iNOS, F13A1, IL1B in primary hepatic macrophages, along with relative quantification of the Western blot. (F) qPCR of *F13a1, Il1b, Cxcl10, Nlrp3, Nos2* mRNA in primary hepatic macrophages. (G,H) Flow cytometry of hepatic non‐parenchymal cells, and the proportion of neutrophils (CD45^+^ Ly6G^+^ Ly6C^lo^) and monocytes (CD45^+^ Ly6G^−^ Ly6C^+^) in CD45^+^ cells. (I,J) Proportion of iNOS^+^ macrophages (CD45^+^ Ly6G^−^ Ly6C^−^ F4/80^+^ CD11B^+^). Data are presented as mean ± standard deviation. For (B,F,H,J), significance was determined by one‐way ANOVA. For (E), significance was determined by Student's *t*‐test. ns = no significance, *p < 0.05, **p < 0.01, ***p < 0.001, ****p < 0.0001. Abbreviations: MASH, metabolic‐associated steatohepatitis; CD, chow diet; CDAHFD, choline‐deficient, L‐amino acid‐defined, high‐fat diet; rAAV, recombinant adeno‐associated virus; NC, negative control; ALT, alanine aminotransferase; AST, aspartate aminotransferase; F13A1, Coagulation Factor XIII Subunit A; MPO, myeloperoxidase, CXCL10, Chemokine (C‐X‐C motif) Ligand 10; IL1B, Interleukin 1 Beta; NLRP3, NACHT, LRR and PYD domains‐containing protein 3; NOS2, Nitric oxide synthase 2; NPC, Non‐Parenchymal Cell; iNOS, Inducible Nitric Oxide Synthase.

To assess the therapeutic potential of targeting F13A1 in vivo, we generated a recombinant adeno‐associated virus vector with preferential liver tropism (rAAV9‐sp146‐C1‐shF13A1) to selectively knock down F13A1 in macrophages [31, 32]. Mice were administered a single tail vein injection of rAAV at week 6 of the CDAHFD‐induced MASH model (Figure 3A). After 2 to 3 weeks of treatment, serum alanine aminotransferase (ALT) and aspartate aminotransferase (AST) levels were significantly reduced (Figure 3B), whereas serum total cholesterol (TC), triglycerides (TG), and fasting glucose remained unchanged (Figure S3A). Liver triglyceride content (Figure S3B) and Oil Red O (Figure S3G) staining further confirmed that lipid accumulation was not substantially altered. These findings indicate that F13A1‐targeted therapy primarily ameliorates hepatic inflammation and hepatocellular injury, with minimal effects on hepatic lipid or glucose metabolism.

Immunofluorescence (Figure 3C) and IHC (Figure S3E) analysis revealed a marked reduction in F13A1^+^ macrophages following rAAV treatment, accompanied by decreased myeloid cell infiltration (Figure S3F). TUNEL staining demonstrated fewer apoptotic hepatocytes in treated mice. In addition, Masson's trichrome staining showed reduced hepatic fibrosis after therapy (Figure 3D; Figure S3C,D), consistent with prior findings.

Isolated hepatic macrophages from rAAV‐treated mice exhibited pronounced suppression of F13A1 expression and significantly lower levels of pro‐inflammatory genes, including *Nos2, Il1b, Cxcl10*, and *Nlrp3*, compared with untreated controls (Figure 3E,F). Flow cytometry revealed a reduced proportion of hepatic neutrophils among CD45^+^ cells, whereas monocyte frequency was unchanged (Figure 3G,H). Among macrophages, the proportion of iNOS^+^ cells was markedly decreased in the rAAV‐treated group (Figure 3I,J), demonstrating attenuation of macrophage pro‐inflammatory activation.

Collectively, these findings demonstrate that suppression of macrophage‐derived F13A1 attenuates macrophage activation and improves hepatic inflammatory injury in MASH mice.

### 2.4 Calcium Enhances F13A1‐PKM2 Binding and Macrophages Activation

**FIGURE 4:**
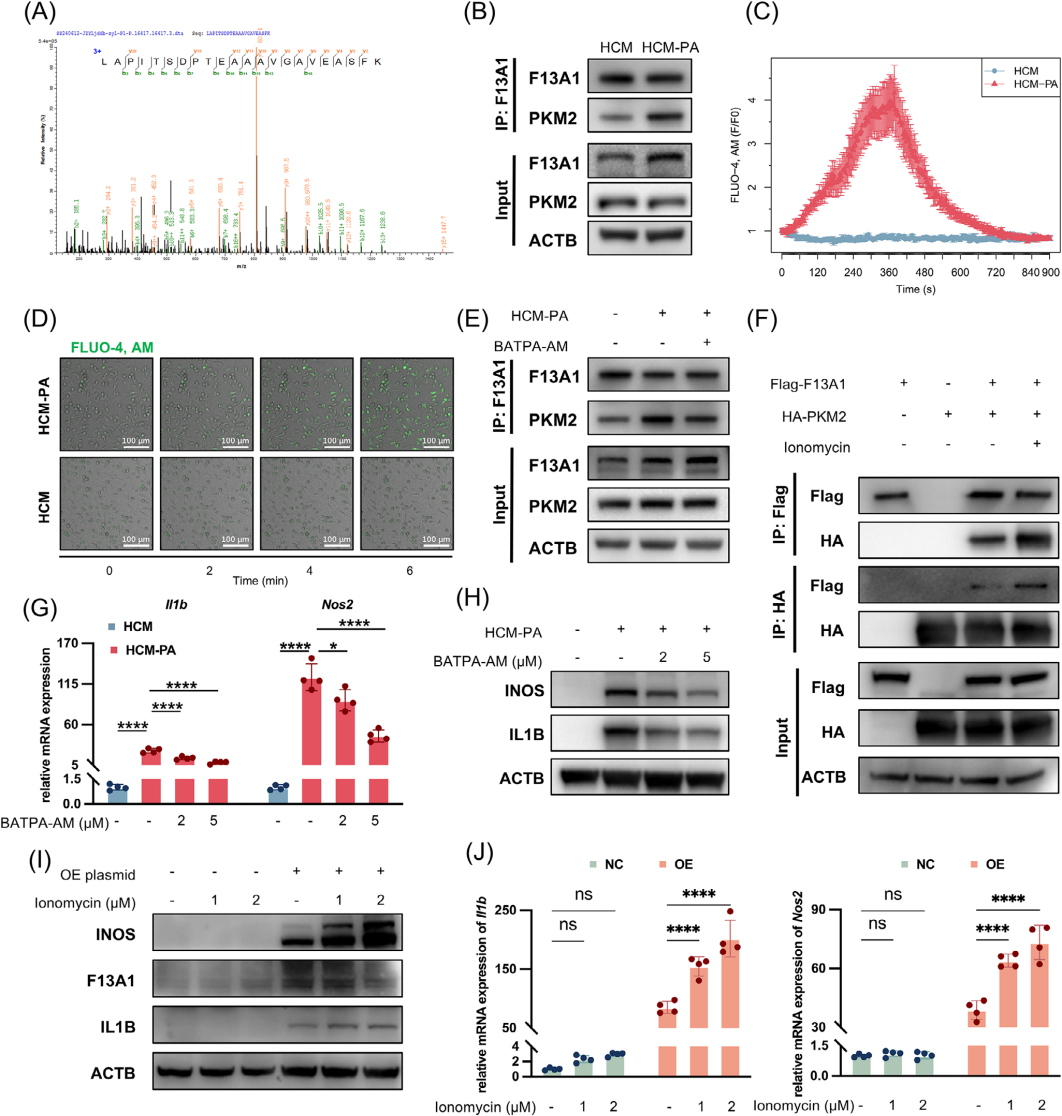
Intracellular Ca^2^⁺ enhances F13A1–PKM2 interaction and F13A1‐mediated macrophage activation. (A) Peptide sequences of PKM2 identified in the F13A1 Co‐IP complex by mass spectrometry. (B) Macrophages were exposed for 24 h to hepatocyte‐conditioned medium (HCM) or to hepatocyte‐conditioned medium derived from palmitic acid‐treated (500 µm, 24 h) hepatocytes (HCM‐PA,), followed by Co‐IP with anti‐F13A1 antibody. (C,D) Confocal imaging of Ca^2^⁺ fluorescence after HCM‐PA stimulation, (C) mean fluorescence intensity from 4 ROIs normalized to baseline; (D) representative images. (E) Macrophages were pretreated with BAPTA‐AM (5 µm, 2 h), followed by HCM‐PA (24 h); Co‐IP was performed with anti‐F13A1 antibody. (F) HEK293T cells were transfected with Flag‐F13A1 and HA‐PKM2 plasmids (24 h), followed by treatment with ionomycin (2 µm, 12 h); Co‐IP was performed with anti‐Flag and anti‐HA antibodies. (G, H) BMDMs were pretreated with BAPTA‐AM (2 or 5 µm, 2 h), followed by HCM‐PA (24 h); gene expression of F13A1, IL1B, and NOS2 was measured by qPCR (G) and Western blotting (H). (I, J) RAW264.7 cells were transfected with F13A1 overexpression plasmid (8 h), then treated with ionomycin (1 or 2 µM, 12 h); Western blot of F13A1, IL1B, iNOS (I) and qPCR of *Il1b, Nos2* (J). Data are presented as mean ± standard deviation. For (G), significance was determined by one‐way ANOVA. For (J) significance was determined by two‐way ANOVA. ns = no significance, *p < 0.05, **p < 0.01, ***p < 0.001, ****p < 0.0001. Abbreviations: PKM2, pyruvate kinase M2 isoform, Co‐IP, co‐immunoprecipitation; ROI, region of interest.; PA, palmitic acid; HCM, hepatocyte‐conditioned medium; HCM‐PA, palmitic acid–treated hepatocyte‐conditioned medium; BMDM, bone marrow‐derived macrophage, F13A1, Coagulation Factor XIII Subunit A; IL1B, Interleukin 1 Beta; NOS2, Nitric oxide synthase 2.

To identify downstream targets of F13A1, we performed mass spectrometry on F13A1 immunoprecipitates from BMDMs. PKM2, an isoform of pyruvate kinase highly expressed in immune cells and known to regulate macrophage activation [20], was identified as a potential interacting partner (Figure 4A). Co‐immunoprecipitation (Co‐IP) and Western blot analyses confirmed the association between F13A1 and PKM2, and this interaction was further strengthened following HCM‐PA stimulation (Figure 4B; Figure S4A).

Previous studies have demonstrated that calcium ions can enhance the intracellular activity of F13A1 [33, 34]. We therefore hypothesized that HCM‐PA may promote F13A1‐PKM2 binding by increasing intracellular Ca^2+^ levels. Using the Ca^2+^ indicator FLUO‐4 AM, we observed a gradual rise in intracellular Ca^2+^ in BMDMs after HCM‐PA treatment (Figure 4C,D). To test whether Ca^2+^ was required for F13A1‐PKM2 association, we applied the cell‐permeable Ca^2+^ chelator BAPTA‐AM. Co‐IP assays showed that chelating intracellular Ca^2+^ markedly attenuated the interaction between F13A1 and PKM2 (Figure 4E; Figure S4B).

We further examined this interaction in HEK293T cells. Co‐transfection of Flag‐F13A1 and HA‐PKM2 resulted in detectable basal binding, and elevating intracellular Ca^2+^ with ionomycin further increased the strength of this association (Figure 4F; Figure S4C).

We next assessed the role of Ca^2+^ in macrophage activation. BAPTA‐AM significantly suppressed HCM‐PA‐induced pro‐inflammatory activation of macrophages (Figure 4G,H; Figure S4D), indicating that Ca^2+^ is required for this response. To assess whether elevated Ca^2+^ could potentiate the pro‐inflammatory effect of F13A1 on macrophages, RAW264.7 cells were transfected with an F13A1 expression plasmid and subsequently exposed to ionomycin. While ionomycin alone failed to trigger pro‐inflammatory activation in RAW264.7 cells, it markedly enhanced the pro‐inflammatory effect of F13A1 overexpression (Figure 4I,J; Figure S4E).

Collectively, these findings identify PKM2 as a downstream effector of F13A1 and demonstrate that elevated intracellular Ca^2+^ enhances F13A1‐PKM2 binding, thereby amplifying macrophage inflammatory activation.

### 2.5 F13A1 Promotes PKM2 Nuclear Translocation and the Warburg Effect in Macrophages Through PKM2 Dimerization

**FIGURE 5:**
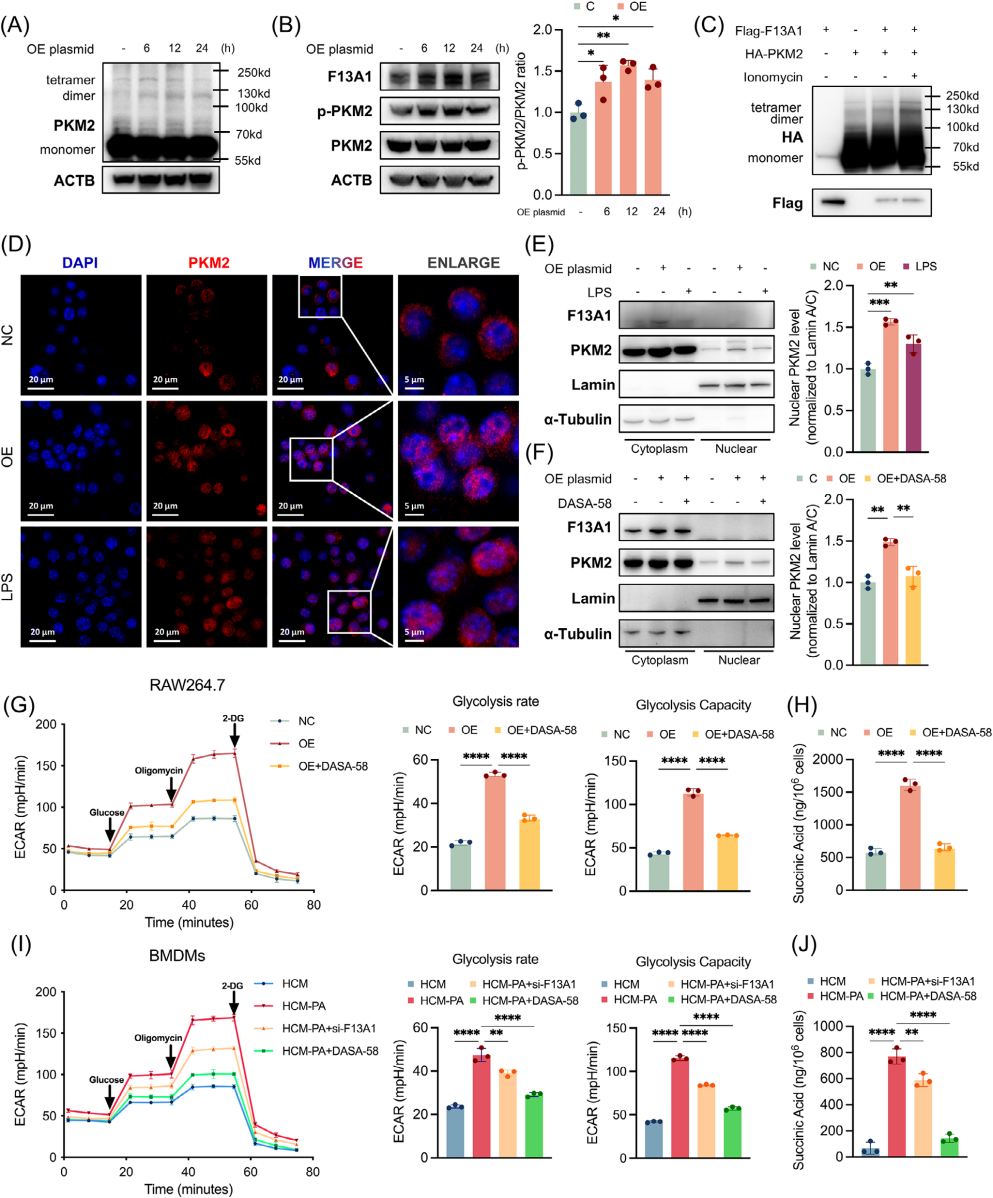
F13A1 promotes PKM2 nuclear translocation and the Warburg effect in macrophages through PKM2 dimerization. (A) RAW264.7 cells were transfected with F13A1 plasmid for 6, 12, or 24 h; Western blot of PKM2 monomer (∼60 kDa), dimer (∼120 kDa), and tetramer (∼240 kDa). (B) Phosphorylation of PKM2 Y105 in RAW264.7 cells after F13A1 overexpression for 6, 12, or 24 h was detected by Western blot, along with relative quantification of p‐PKM2 normalized to total PKM2. (C) HEK293T cells were transfected with Flag‐F13A1 and HA‐PKM2 plasmids (24 h), followed by treatment with ionomycin (2 µm, 12 h), Western blot of HA‐PKM2 monomer (∼60 kDa), dimer (∼120 kDa), and tetramer (∼240 kDa). (D,E) Cytoplasmic and nuclear distribution of PKM2 in RAW264.7 cells after F13A1 overexpression (12 h) or LPS stimulation (12 h), detected by confocal microscopy (D) and Western blot, along with relative quantification of nuclear PKM2 (E). (F) RAW264.7 cells transfected with F13A1 plasmid and treated with DASA‐58 (50 µm) 12 h; cytoplasmic and nuclear distribution of PKM2 detected by Western blot along with relative quantification of nuclear PKM2. (G,H) RAW264.7 cells were transfected with F13A1 plasmid and were treated with DASA‐58 (50 µm) 12 h; glycolytic rate and capacity of RAW264.7 cells were measured by real‐time recording of extracellular acidification rates (ECAR) after successive injection of glucose, oligomycin, and 2‐DG (G). Intracellular succinate was detected by LC‐MS/MS (H). (I,J) BMDMs were treated with HCM or HCM‐PA (12 h), followed by DASA‐58 (50 µm, 12 h) or were pretreated with si‐F13A1 for 12 h; glycolytic rate, glycolytic capacity, (I) and intracellular succinate (J) were measured by the same approaches (J). Data are presented as mean ± standard deviation. For (B) and (E–J), significance was determined by one‐way ANOVA. ns = no significance, *p < 0.05, **p < 0.01, ***p < 0.001, ****p < 0.0001. Abbreviations: PKM2, pyruvate kinase M2 isoform, F13A1, Coagulation Factor XIII Subunit A; ECAR, extracellular acidification rates; LC‐MS/MS, liquid chromatography‐tandem mass spectrometry; 2‐DG, 2‐Deoxy‐D‐glucose; HCM, hepatocyte‐conditioned medium; HCM‐PA, palmitic acid–treated hepatocyte‐conditioned medium.

Previous studies have reported that PKM2 exists in both tetrameric and dimeric forms. The tetrameric form primarily exerts kinase activity to facilitate glycolysis, whereas the dimeric form can translocate into the nucleus, interact with HIF1A, and enhance the binding of HIF1A to its target genes [20, 24]. We therefore hypothesized that F13A1 may promote macrophage activation by shifting PKM2 toward its dimeric form. To validate this hypothesis, we overexpressed F13A1 in RAW264.7 cells and observed a marked increase in PKM2 dimerization (Figure 5A). Given that phosphorylation at Y105 is indicative of PKM2 dimer formation, we assessed levels of phosphorylated Y105‐PKM2 and found that F13A1 overexpression also promoted Y105 phosphorylation of PKM2 (Figure 5B). In HEK293T cells co‐transfected with Flag‐F13A1 and HA‐PKM2, co‐expression induced detectable HA‐PKM2 dimer formation, and increasing intracellular Ca^2+^ with ionomycin further augmented PKM2 dimerization (Figure 5C), providing direct evidence that F13A1 promotes PKM2 dimerization in a Ca^2+^‐responsive manner.

We next examined PKM2 nuclear translocation. Immunofluorescence staining demonstrated that F13A1 overexpression increased PKM2 nuclear import, with LPS serving as a positive control (Figure 5D). Cytoplasmic and nuclear fractionation corroborated enhanced nuclear localization of PKM2 in cells overexpressing F13A1 (Figure 5E). In BMDMs, HCM‐PA stimulation similarly promoted PKM2 nuclear translocation, and this effect was partially suppressed by F13A1 knockdown, indicating that F13A1 contributed to HCM‐PA‐induced PKM2 nuclear import (Figure S5). Moreover, treatment with DASA‐58, a PKM2 activator that facilitates PKM2 tetramerization, effectively inhibited F13A1‐driven PKM2 nuclear translocation (Figure 5F).

Dimeric PKM2 also induces metabolic reprogramming in macrophages and triggers the Warburg effect, which in turn further promotes macrophage activation [20]. Consistent with this, F13A1 overexpression significantly increased the glycolytic rate and glycolytic capacity of RAW264.7 cells, whereas DASA‐58 reversed these effects (Figure 5G). A metabolic consequence of this shift is the accumulation of succinate, a pro‐inflammatory metabolite that stabilizes HIF1A and amplifies macrophage activation [35]. F13A1 overexpression led to pronounced succinate accumulation, which was similarly corrected by DASA‐58 (Figure 5H). In BMDMs, HCM‐PA promoted both glycolysis and succinate accumulation, and these effects were attenuated by either F13A1 knockdown or DASA‐58 treatment (Figure 5I,J).

Together, these findings demonstrate that F13A1 promotes PKM2 dimerization, nuclear translocation, and glycolytic reprogramming.

### 2.6 F13A1 Activates Macrophages Through the PKM2/HIF1A Axis

**FIGURE 6:**
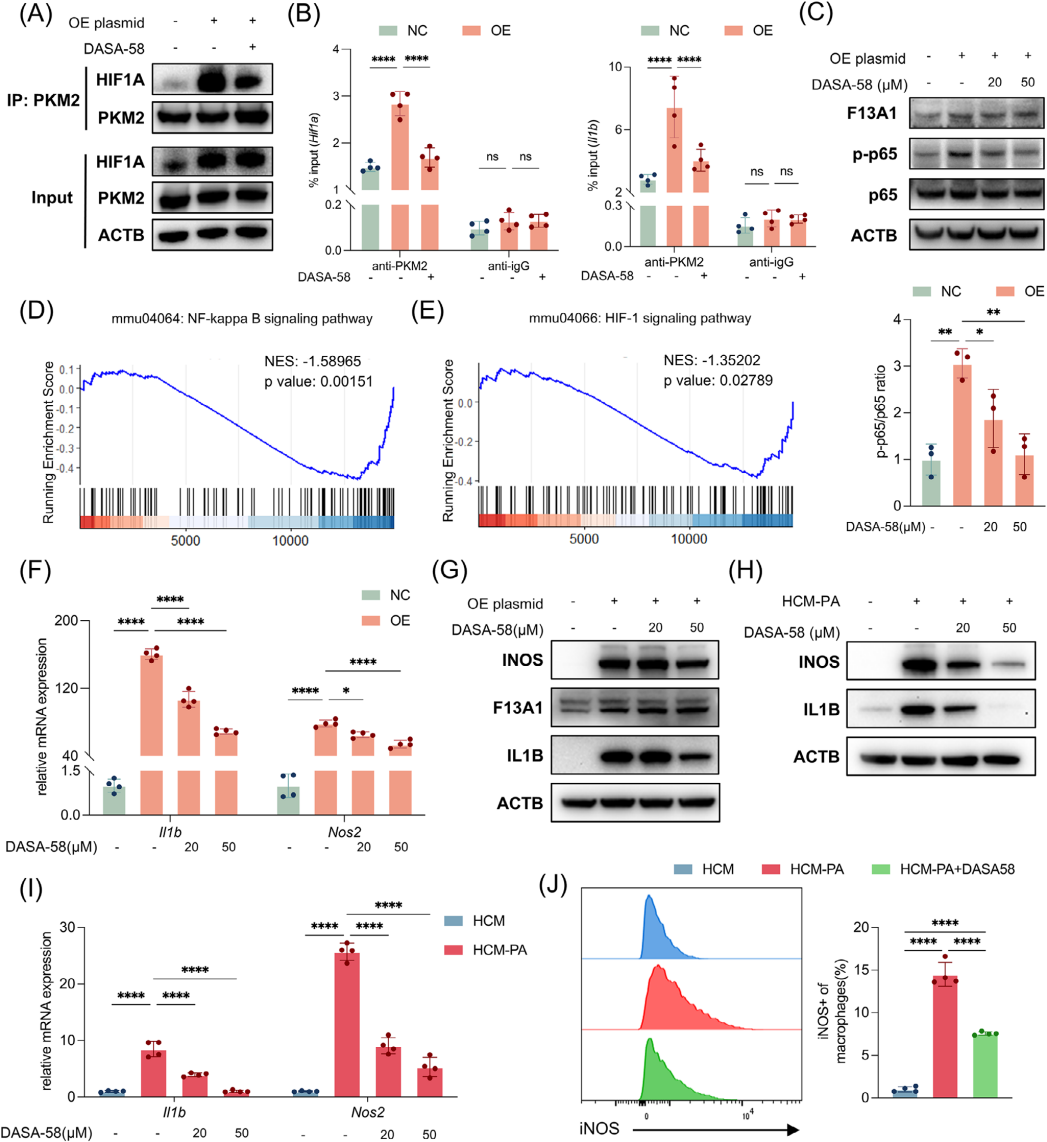
F13A1 activates macrophages through the PKM2/HIF1A axis. (A, B) RAW264.7 cells were transfected with F13A1 plasmid and were treated with DASA‐58 (50 µm) 12 h; PKM2–HIF1A interaction detected by Co‐IP with anti‐PKM2 antibody (A); PKM2 binding to *Il1b* and *Hif1a* promoters detected by ChIP‐PCR with anti‐PKM2 antibody (B). (C) RAW264.7 cells were transfected with F13A1 plasmid and were treated with DASA‐58 (20 or 50 µm, 12 h); phosphorylation of NF‐κB(p65) detected by Western blot, along with relative quantification of p‐p65 normalized to total p65. (D,E) GSEA of BMDMs after F13A1 knockdown (24 h); NF‐κB and HIF‐1 signaling pathways were suppressed. (F,G) RAW264.7 cells were transfected with F13A1 plasmid and treated with DASA‐58 (20 or 50 µm, 24 h); mRNA levels of *Il1b*, *Nos2* (F), and protein levels of F13A1, IL1B, iNOS (G). (H,I) BMDMs were treated with HCM‐PA ± DASA‐58 (20 or 50 µm, 24 h); gene expression of IL1B and NOS2 was measured by Western blotting (H) and qPCR (I). (J) BMDMs were treated with HCM‐PA ± DASA‐58 (50 µm, 24 h); flow cytometry of iNOS⁺ macrophages. Data are presented as mean ± standard deviation. For (B), significance was determined by two‐way ANOVA. For (C,F,I,J), significance was determined by one‐way ANOVA. ns = no significance, *p < 0.05, **p < 0.01, ***p < 0.001, ****p < 0.0001. Abbreviations: GSEA, Gene Set Enrichment Analysis; NF‐κB, nuclear factor κB; HIF1A, hypoxia‐inducible factor 1‐alpha; ChIP‐PCR, chromatin immunoprecipitation PCR; PKM2, pyruvate kinase M2 isoform, F13A1, Coagulation Factor XIII Subunit A; IL1B, Interleukin 1 Beta; iNOS, Inducible Nitric Oxide Synthase; HCM, hepatocyte‐conditioned medium; HCM‐PA, palmitic acid–treated hepatocyte‐conditioned medium.

Next, we examined whether F13A1 activates macrophages through the PKM2/HIF1A axis as expected. First, we tested whether F13A1 overexpression facilitated PKM2 binding to HIF1A. Co‐IP assays revealed enhanced PKM2‐HIF1A interaction following F13A1 overexpression, which was attenuated by DASA‐58 treatment (Figure 6A; Figure S6A). Previous studies have shown that PKM2 cooperates with HIF1A to induce IL1B transcription and can also enhance HIF1A expression through a positive feedback loop [20, 24]. Consistent with these findings, chromatin immunoprecipitation (ChIP) assays revealed that F13A1 overexpression increased PKM2 binding to the *Il1b* and *Hif1a* promoters, and this effect was similarly suppressed by DASA‐58 (Figure 6B).

Additionally, PKM2 has also been reported to activate the nuclear factor κB (NF‐κB) signaling pathway, a central regulator of macrophage activation. Notably, the NF‐κB pathway can also function as an upstream activator of HIF1A [36]. In agreement with this, F13A1 overexpression increased phosphorylation of NF‐κB(p65), whereas DASA‐58 abolished this response (Figure 6C). Reanalysis of transcriptomic profiles from BMDMs with F13A1 knockdown further showed coordinated downregulation of both the NF‐κB and HIF‐1 pathways (Figure 6D,E).

Finally, we tested whether DASA‐58 could reverse macrophage activation driven by elevated F13A1. DASA‐58 significantly reduced F13A1‐induced iNOS and IL1B expression in RAW264.7 cells (Figure 6F,G; Figure S6B), demonstrating that PKM2 is a key downstream effector of F13A1. Similarly, DASA‐58 suppressed HCM‐PA‐induced expression of IL1B and iNOS in BMDMs (Figure 6H,I; Figure S6C) and reduced the proportion of pro‐inflammatory macrophages, as confirmed by flow cytometry (Figure 6J).

Overall, F13A1 activates macrophages through the PKM2/HIF1A pathway, whereas treatment with the PKM2 activator DASA‐58 effectively suppresses macrophage activation.

### 2.7 S1P Mediates Hepatocyte‐Induced Macrophages Calcium Signaling and F13A1 Expression

**FIGURE 7:**
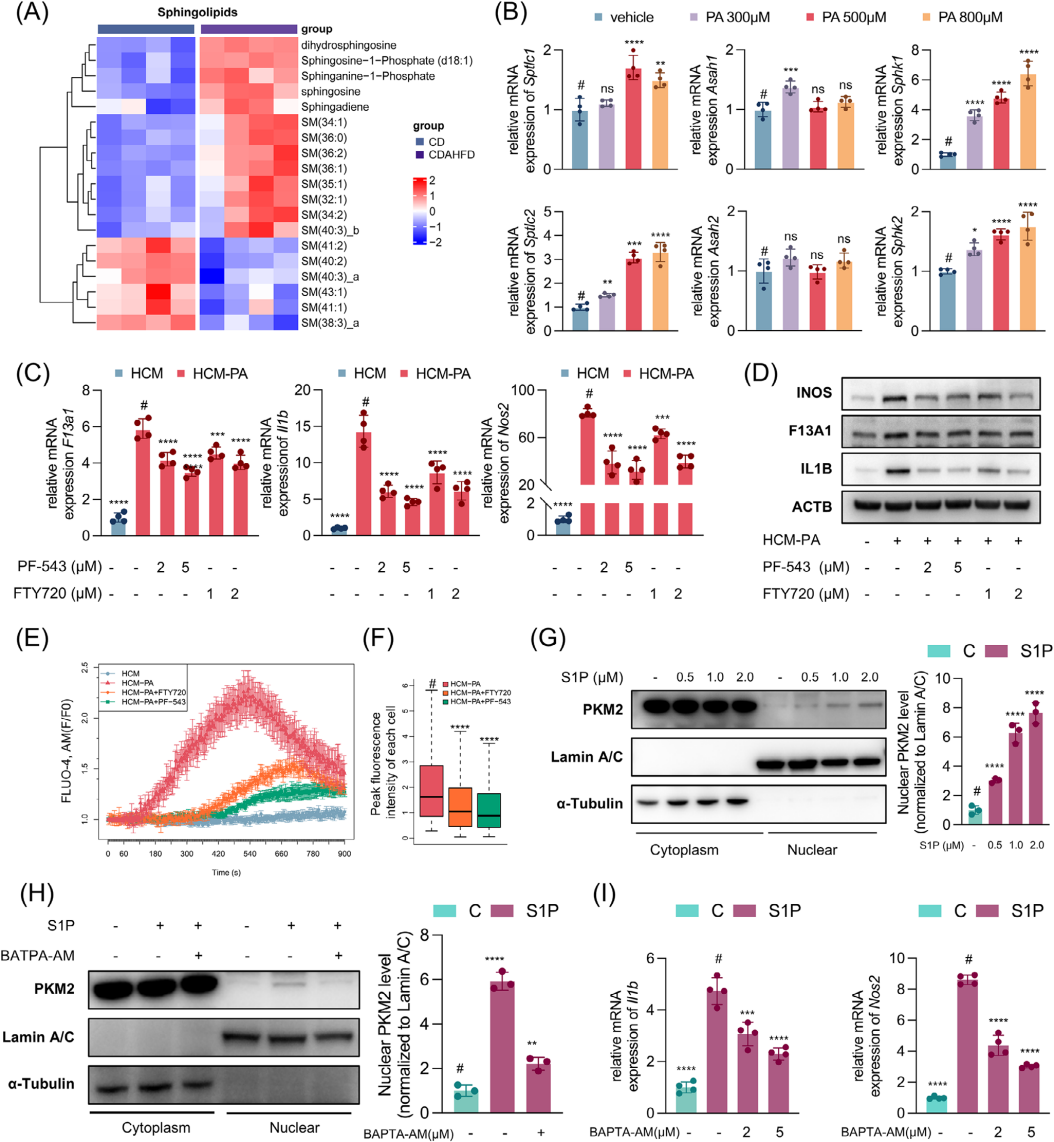
S1P mediates hepatocyte‐induced F13A1 expression and intracellular Ca^2^⁺ elevation in macrophages under lipid overload. (A) Differential hepatic sphingolipids between CDAHFD‐fed mice (8 weeks) and controls (FDR<0.05). (B) Primary hepatocytes were treated with palmitic acid (300, 500, 800 µm, 24 h) or vehicle control; expression of key enzymes in the S1P synthesis pathway: Sptlc1 and Sptlc2 (de novo ceramide synthesis), Asah1 and Asah2 (sphingosine synthesis), Sphk1 and Sphk2 (S1P synthesis). (C,D) Conditioned medium was collected from hepatocytes treated with vehicle or palmitic acid (500 µm, 24 h) (HCM/HCM‐PA); BMDMs were treated with HCM or HCM‐PA for 24 h, with or without PF543 (2 or 5 µm) or FTY720 (1 or 2 µm) to inhibit S1P synthesis or function; mRNA levels (C) and protein levels (D) of F13A1, IL1B, iNOS. (E,F) Hepatocytes were treated with palmitic acid (500 µm, 24 h) to generate HCM‐PA, with or without PF‐543 (5 µm), then the supernatant was collected to stimulate BMDMs (HCM‐PA+PF‐543); or HCM‐PA was used to stimulate BMDMs with/without FTY720 pretreatment (2 µm, 2 h) to block S1P function (HCM‐PA+FTY720); intracellular Ca^2+^ fluorescence was assessed by confocal microscopy; mean fluorescence intensity from four ROIs was normalized to baseline (E) and peak fluorescence intensity per cell quantified by CellProfiler (F). (G) stimulation of BMDMs with S1P (0.5, 1, 2 µm) for 2 h; western blot of cytoplasmic and nuclear PKM2 with relative quantification. (H) BMDMs were pretreated with BAPTA‐AM (5 µm, 2 h), followed by S1P (2 µm) stimulation (3 h), western blot of cytoplasmic and nuclear PKM2 with relative quantification. (I) BMDMs were pretreated with BAPTA‐AM (2, 5 µm, 2 h), followed by S1P (2 µM) stimulation (3 h); qPCR of *Il1b* and *Nos2* mRNA. Data are presented as mean ± standard deviation (B, C, E, G, H, I) or as median with interquartile range (F). For (B,C,I), significance was determined by one‐way ANOVA. For (F), significance was determined by the Kruskal–Wallis test. # indicates control group. ns = no significance, *p < 0.05, **p < 0.01, ***p < 0.001, ****p < 0.0001. Abbreviations: CDAHFD, choline‐deficient, L‐amino acid‐defined, high‐fat diet; S1P, sphingosine‐1‐phosphate; SPTLC, serine palmitoyltransferase; ASAH, acid ceramidase; SPHK, sphingosine kinase; HCM‐PA, palmitic acid–treated hepatocyte‐conditioned medium; BMDM, bone marrow–derived macrophage; F13A1, Coagulation Factor XIII Subunit A; IL1B, Interleukin 1 Beta; iNOS, Inducible Nitric Oxide Synthase; ROI, Region of Interest.

To identify the mediators responsible for hepatocyte‐macrophage crosstalk, we performed lipidomic profiling of livers from MASH mice. Compared with controls, the major lipid classes elevated in MASH included fatty acids, glycerophospholipids, and sphingolipids (Figure S7A). Among sphingolipids, both sphingosine and its phosphorylated derivative, sphingosine‐1‐phosphate (S1P), were markedly increased (Figure 7A). S1P is a well‐established bioactive lipid mediator; prior studies have shown that palmitate‐stressed hepatocytes release S1P‐enriched extracellular vesicles that promote macrophage chemotaxis, and that S1P receptor signaling can trigger intracellular Ca^2+^ release via the PLC‐IP_3_ pathway [37, 38, 39]. These findings suggested a potential role for S1P in hepatocyte‐induced macrophage activation.

We first examined the expression of S1P biosynthetic enzymes under lipid overload. Palmitate‐stimulated hepatocytes exhibited significant upregulation of serine palmitoyltransferase (SPTs), the rate‐limiting enzymes for *de novo* ceramide synthesis, and sphingosine kinases (SPHKs), which convert sphingosine to S1P. Among these, Sphk1 displayed the most pronounced increase (Figure 7B). To functionally test the role of S1P, we introduced a selective SPHK1 inhibitor (PF‐543) and an S1P antagonist (FTY720) in the hepatocyte‐conditioned medium co‐culture system to inhibit S1P synthesis or signaling. Both compounds markedly reduced pro‐inflammatory polarization and F13A1 expression in BMDMs (Figure 7C,D; Figure S7C) and attenuated Ca^2+^ signaling induced by hepatocyte supernatant (Figure 7E,F; Figure S7B). These results indicate that hepatocyte‐derived S1P contributes to macrophage activation and Ca^2+^ signaling.

We next evaluated whether S1P regulates PKM2 signaling in a Ca^2+^‐dependent manner. Direct stimulation of BMDMs with S1P promoted PKM2 nuclear import (Figure 7G), whereas BAPTA‐AM effectively blocked this effect (Figure 7H). S1P alone induced *Il1b, Nos2*, and *F13a1* expression, although the magnitude was modest and *Il1b* induction was transient, peaking early and declining after 12 h (Figure S7D). BAPTA‐AM suppressed S1P‐induced *Il1b* and *Nos2* expression, demonstrating that S1P‐mediated macrophage activation requires intracellular Ca^2+^ (Figure 7I). Furthermore, F13A1 knockdown also attenuated S1P‐induced macrophage activation (Figure S7E).

Collectively, these findings demonstrate that under lipid overload, hepatocytes activate macrophage Ca^2+^ signaling and induce F13A1 expression through S1P. In turn, S1P promotes PKM2 nuclear translocation and macrophage pro‐inflammatory activation in a Ca^2+^‐dependent manner.

### 2.8 PKM2 Targeted Therapy Alleviates Hepatic Inflammation in MASH

**FIGURE 8:**
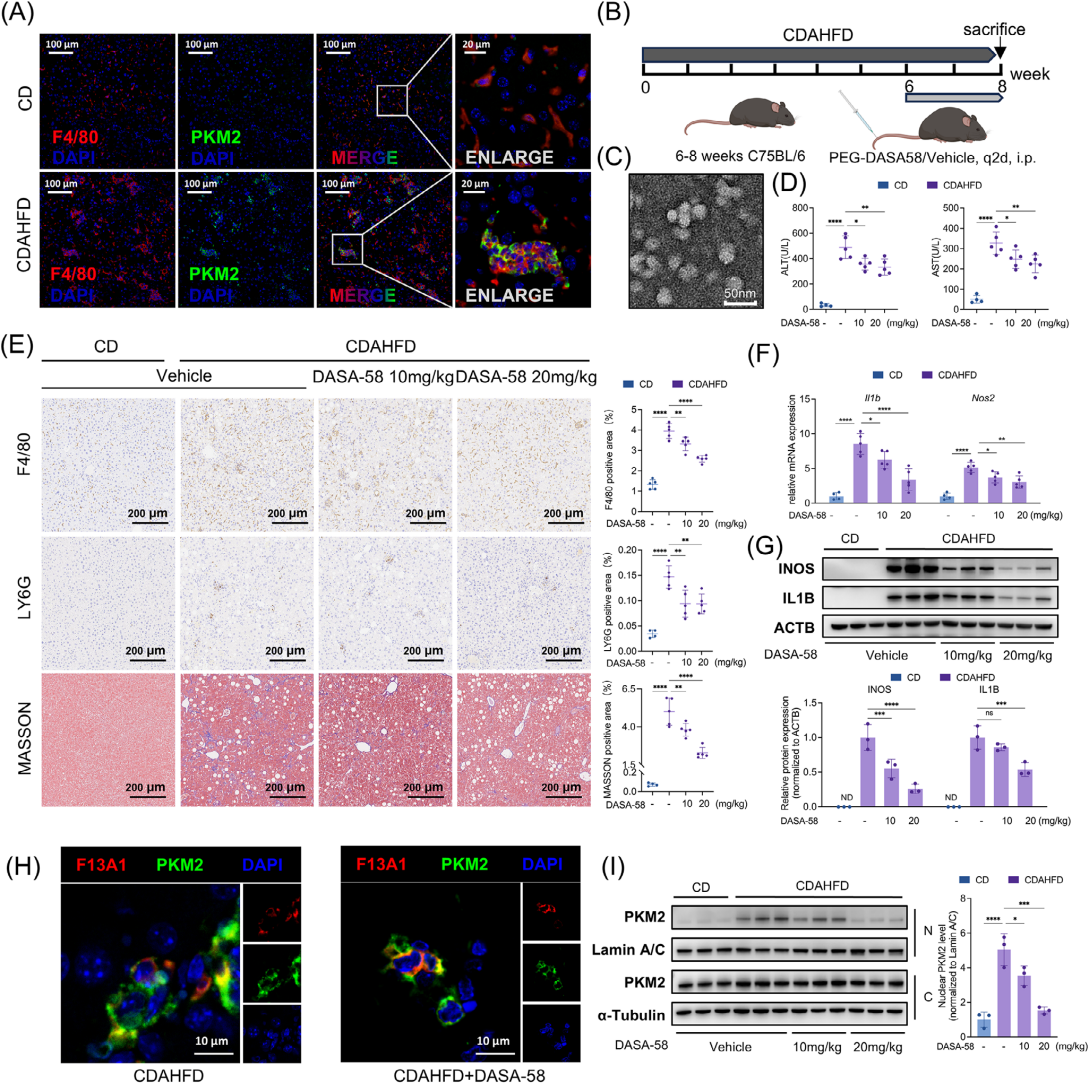
PEG‐PLA micelle‐encapsulated DASA‐58 alleviates hepatic inflammation in MASH mice and suppresses macrophage PKM2 nuclear translocation and pro‐inflammatory gene expression. (A) Immunofluorescence staining of F4/80 (red), F13A1 (green), and nuclei (DAPI, blue) in CD or CDAHFD‐fed mouse liver. (B) CDAHFD‐fed mice were treated by intraperitoneal injection of PEG‐PLA–DASA‐58 (10 or 20 mg/kg) or vehicle at week 6; blood and liver were collected after 2 weeks treatment. (C) Transmission electron microscopy of PEG‐PLA–DASA‐58 nanoparticles; mean diameter ∼30 nm. (D) Serum ALT and AST after 2 weeks of treatment. (E) Immunohistochemistry of F4/80 and Ly6G, and Masson staining of liver; quantification of positive staining area. (F,G) Primary hepatic macrophages were isolated from each group; gene expression of IL1B, NOS2, was measured by qPCR (F) and Western blotting, along with relative quantification of the Western blot (G). (H) Immunofluorescence of PKM2 (green) and F13A1 (red) in liver from CDAHFD and treated mice; nuclei counterstained with DAPI (blue). (I) Cytoplasmic and nuclear distribution of PKM2 in primary hepatic macrophages was detected by Western blot, along with relative quantification of nuclear PKM2. Data are presented as mean ± standard deviation. For (D,E,F,G,I), significance was determined by one‐way ANOVA. ND = not detected, ns = no significance, *p < 0.05, **p < 0.01, ***p < 0.001, ****p < 0.0001. Abbreviations: MASH, Metabolic‐Associated Steatohepatitis; PEG‐PLA, Polyethylene Glycol‐Polylactic Acid; CD, chow diet; CDAHFD, choline‐deficient, L‐amino acid‐defined; ALT, Alanine Aminotransferase; AST, Aspartate Aminotransferase; PKM2, pyruvate kinase M2 isoform; IL1B, Interleukin 1 Beta; iNOS, Inducible Nitric Oxide Synthase.

Previous studies have shown that PKM2 is preferentially expressed in macrophages compared to hepatocytes and is further upregulated in the MASH microenvironment [40]. We confirmed this observation by immunofluorescence analysis (Figure 8A). Based on this characteristic and the potent inhibitory effect of DASA‐58 on macrophage activation, we sought to evaluate the therapeutic potential of DASA‐58 in alleviating hepatic inflammation in MASH mice. In preliminary experiments, we observed that DASA‐58 exhibited poor water solubility and potential hepatotoxicity. To overcome these drawbacks, we utilized a PEG‐PLA based micellar drug delivery system to encapsulate DASA‐58 (PEG‐PLA‐DASA‐58) (Figure 8C), which provides improved water solubility and prolonged drug half‐life, thereby allowing for dose reduction. Next, PEG‐PLA‐DASA‐58 was administered after 6 weeks of CDAHFD diet (Figure 8B). Following two‐week treatment, a significant reduction in serum ALT/AST levels was observed (Figure 8D), indicating an improvement in hepatic inflammation. IHC staining revealed decreased infiltration of inflammatory cells such as neutrophils and macrophages in liver tissue. Masson's staining further demonstrated an improvement in hepatic fibrosis (Figure 8E).

Next, we investigated the expression of pro‐inflammatory genes in hepatic macrophages. As expected, expression levels of *Nos2, Il1b, Il6, and Tnf* were decreased upon treatment (Figure 8F; Figure S8A), and Western blotting further confirmed reductions in iNOS and IL1B protein levels (Figure 8G). Immunofluorescence analysis showed strong colocalization between F13A1 and PKM2, as well as evident nuclear translocation of PKM2 in hepatic macrophages in MASH mice. Notably, the number of cells with nuclear PKM2 was reduced after DASA‐58 treatment (Figure 8H; Figure S8B). This finding was corroborated by cytoplasmic/nuclear fractionation assays performed on isolated liver macrophages following DASA‐58 treatment (Figure 8I).

Collectively, these results highlight the critical contribution of PKM2 nuclear translocation to hepatic macrophage activation during MASH progression and underscore the promising therapeutic potential of targeting PKM2 with DASA‐58 for MASH intervention.

## 3 Discussion

Originally identified as a fibrin‐stabilizing factor in the 1940s, F13A1 has long been viewed primarily as a terminal enzyme in the coagulation cascade. However, growing evidence indicates that F13A1 also performs important non‐coagulant functions, particularly within myeloid cells [16, 17]. In the present study, we uncovered a previously unrecognized role for F13A1 in driving macrophage activation during MASH progression. We showed that F13A1 promotes PKM2 dimerization and enhances HIF1A transactivation, thereby linking a classical coagulation factor to a central metabolic‐inflammatory pathway. Notably, a study by AbdAlla et al. reported that F13A1 facilitates AT1 receptor dimerization in monocytes [34], supporting the notion that stabilizing protein dimer formation may represent a broader intracellular function of F13A1.

The origin and induction mechanisms of F13A1^+^ macrophages remain intriguing and are not yet fully understood. Griffin et al. found that selectively deleting F13A1 in either myeloid cells or platelets markedly reduced plasma F13A1 levels, whereas platelet‐deficient mice showed no change in circulating F13A1 [12]. A subsequent study further demonstrated that F13A1 is predominantly expressed by myeloid cells [11]. These findings support the notion that F13A1 may serve as a marker of monocyte‐derived macrophages. The increased proportion of F13A1^+^ macrophages observed in our study is therefore likely to reflect enhanced infiltration of monocyte‐derived macrophages in the MASH liver. Despite these insights, the upstream regulatory mechanisms controlling F13A1 expression remain poorly characterized. Griffin et al. reported that F13A1 expression increases during the differentiation of myeloid progenitors into granulocyte‐macrophage progenitors [12], and in vitro studies have shown that alternative macrophage activation [41] and oxidized LDL‐induced foam cell formation [10] also elevate F13A1 expression. However, the signaling pathways governing these responses remain unclear. Our findings identified hepatocyte‐derived S1P as a key factor that sustains and promotes F13A1 expression in macrophages under lipid‐overloaded conditions, although the precise mechanisms through which S1P regulates F13A1 transcription require further investigation.

S1P signaling has been strongly implicated in the progression of steatohepatitis [42, 43] and liver fibrosis [44, 45], with SPHK1 recognized as a major enzyme responsible for pathological S1P accumulation [44, 46, 47, 48]. Palmitic acid has also been reported to stimulate S1P synthesis in hepatocytes [49]. In addition, S1P serves as a “find‐me” signal released by apoptotic cells [50], suggesting that the abundance of apoptotic cells in MASH may further elevate hepatic S1P levels. Pharmacological inhibition of S1P with antagonists such as FTY720 has been reported to attenuate hepatic steatosis and inhibit the progression of liver fibrosis [51]. Consistent with these findings, our study highlights a pivotal role for S1P in steatohepatitis and further revealed that S1P promotes the F13A1 signaling pathway through two complementary mechanisms: activation of intracellular Ca^2+^ signaling and induction of F13A1 expression in macrophages. Intriguingly, since FTY720 (fingolimod) is also a well‐established immunosuppressant, it is conceivable that in specific clinical contexts, such as liver transplantation using steatotic donor livers [52], FTY720 could serve as a preferred therapeutic option offering dual benefits—preventing graft rejection while simultaneously ameliorating MASH. This concept warrants further investigation in future translational and clinical studies.

PKM2 serves as a pivotal metabolic regulator in macrophages, integrating metabolic cues with inflammatory signaling. Its expression and activation are triggered by diverse metabolic and inflammatory stimuli‐including LPS [20], oxidized LDL [53], and cholesterol crystals [54]. Because PKM2 is an alternatively spliced isoform of the PKM gene, its abundance is controlled at both the transcriptional and splicing levels. HIF1A can transcriptionally induce PKM2 [24], whereas the mTOR/HIF1A/Myc/hnRNPs pathway regulates its alternative splicing toward the PKM2 isoform [55]. Importantly, PKM2 is highly enriched in macrophages and is nearly undetectable in normal hepatocytes, underscoring its appeal as a selective therapeutic target for MASH [40]. Several PKM2‐targeting compounds have already shown therapeutic benefit in murine models of steatohepatitis, reducing hepatic steatosis, inflammation, and fibrosis [23, 56, 57]. Consistent with these findings, PEG‐PLA‐encapsulated DASA‐58 in our study further enhanced macrophage targeting and produced robust therapeutic effects, collectively reinforcing PKM2 as a compelling and tractable target for MASH intervention.

This study has several limitations that should be acknowledged. First, although our data demonstrated that hepatocyte‐derived S1P induces macrophage activation and enhances F13A1 expression, the precise downstream signaling events remain to be fully delineated. Second, although F13A1^+^ macrophages were initially identified in human liver samples, most mechanistic studies were conducted in murine systems. Thus, potential species‐specific differences should be carefully considered when translating these findings to human MASH. Finally, the possibility of off‐target effects with DASA‐58 and rAAV treatment cannot be completely ruled out.

In summary, this study identified the massive infiltration of F13A1⁺ inflammatory macrophages in the liver during MASH, and established F13A1 as a previously unrecognized driver of macrophage classical activation. Mechanistically, we demonstrated that F13A1 activates the PKM2/HIF1A pathway in macrophages, a process amplified by hepatocyte‐derived S1P, thereby connecting hepatocyte metabolic stress to macrophage inflammatory responses in MASH. Furthermore, pharmacological activation of PKM2 effectively disrupted this pathway, highlighting PKM2 as a promising therapeutic target and supporting the translational potential of PKM2‐directed interventions for MASH.

## 4 Methods

### 4.1 Human Liver Specimens and Ethics

Human liver tissue samples were collected from patients undergoing partial hepatectomy for benign diseases at ‌the First Affiliated Hospital, Zhejiang University School of Medicine‌ (Hangzhou, China). Healthy liver tissues (n = 10) and MASH liver tissues (n = 10) were obtained with informed consent from all participants. The study protocol was approved by the Ethics Committee of ‌the First Affiliated Hospital, Zhejiang University School of Medicine (Ethics approval number: IIT20210329B‐R1), and all procedures conformed to the principles outlined in the Declaration of Helsinki (2013) and Declaration of Istanbul (2018). Written consent was obtained from each patient prior to sample collection

### 4.2 Animal Models and In Vivo Interventions

Male C57BL/6J mice (6‐8 weeks) were maintained under SPF conditions with free access to food and water. MASLD was induced by feeding a high‐fat diet (HFD; Research Diets, D12492), whereas MASH was induced using a choline‐deficient, amino acid‐defined, high‐fat diet (CDAHFD; Research Diets, A06071302). For macrophage‐specific F13A1 silencing, a recombinant adeno‐associated virus serotype 9 (rAAV9) encoding F13A1 shRNA under a macrophage‐specific promoter sp146‐C1 [31, 32] (rAAV9‐sp146‐C1‐shF13A1, 2 × 10^11^vg/mouse; Hanbio Biotechnology, Shanghai, China) was administered by tail‐vein injection at week 6 of CDAHFD feeding. The antisense shRNA sequence was 5′‐CTTGTATGTGATGGACAA‐3’. Blood samples were collected 2 weeks post‐injection, and mice were sacrificed at week 3. For PKM2 pharmacologic activation, DASA‐58 (MCE, HY‐19330) was encapsulated in PEG–PLA micelles to improve solubility and stability. PEG‐PLA–DASA‐58 (10 or 20 mg/kg) was administered by daily intraperitoneal injection for 2 weeks starting at week 6 of CDAHFD feeding. At sacrifice, blood and liver samples were collected for biochemical, histological, and molecular analyses. All animal experiments were approved by ZJU‐Laboratory Animal Welfare and Ethics Review Committee (Ethics approval number: ZJU20250892).

### 4.3 Preparation of DASA‐58‐Loaded PEG‐PLA Micelles

DASA‐58‐loaded PEG‐PLA micelles were prepared using a solvent emulsification–evaporation approach as previously described [58]. Briefly, 30 mg PEG2000‐PLA2000 (Tanshtech, 80010401–2000) and 3 mg DASA‐58 were dissolved in 1 mL ethyl acetate (Aladdin, E116142). The organic phase was slowly injected into 5 mL deionized water, followed by sonication to generate an oil‐in‐water emulsion. The solvent was evaporated under reduced pressure for 10 min, and unencapsulated drug was removed by centrifugation (3000 rpm, 10 min). Micelle size and zeta potential were assessed by dynamic light scattering (DLS) using a Malvern Zetasizer Nano ZS90, yielding a mean hydrodynamic diameter of 97.38 ± 20.06 nm and a zeta potential of −21.17 ± 2.66 mV. Transmission electron microscopy (TEM) confirmed spherical morphology with ∼30 nm diameter (Figure 8C).

### 4.4 Public Single‐Nucleus RNA Sequencing and Bulk RNA‐Sequencing Analysis

Four publicly available snRNA‐seq datasets (GSE212046, GSE174748 [25], GSE185477 [26], GSE189175 [27]) were analyzed using Seurat (v5.2.1) in R (v4.3.1). Standard workflows—quality control, normalization, dimensionality reduction, clustering, and UMAP visualization—were applied. Batch effects were corrected using Harmony (v1.2.3). Cells with potential ambient RNA contamination were identified and removed. The GSE135251 [28] dataset included 216 normal and fatty liver samples, each accompanied by respective Non‐Alcoholic Steatohepatitis Activity Score (NAS). The GSE167523 [29] dataset comprised 98 liver samples pathologically diagnosed as simple steatosis or MASH. In the GSE138778 [30], CD‐fed and MCD‐fed mice were subjected to flow cytometry sorting to isolate embryonic‐derived Kupffer cells and monocyte‐derived Kupffer cells, followed by transcriptome sequencing.

### 4.5 Calcium Imaging

Intracellular Ca^2+^ levels were measured using FLUO‐4, AM (Thermo Fisher Scientific, F14201). BMDMs were loaded with 4 µM FLUO‐4, AM in HBSS for 30 min at 37°C, washed, and incubated in dye‐free HBSS. Imaging was performed using a Leica STELLARIS 5 confocal system (excitation 494 nm; emission 516 nm). Mean fluorescence intensity per cell was quantified using CellProfiler (v4.2.5) with automated segmentation.

### 4.6 Primary Cell Isolation and Cell Line

Primary hepatocytes and hepatic macrophages were isolated via in situ liver perfusion with collagenase IV followed by Percoll (cytiva, 17089101) density gradient centrifugation. Hepatic macrophages were further purified with Anti‐F4/80 MicroBeads UltraPure (Miltenyi Biotec, 130‐110‐443). BMDMs were generated by culturing bone marrow cells in RPMI‐1640 supplemented with 10% FBS and macrophage colony‐stimulating factor (M‐CSF, ABclonal, RP01216, 20 ng/mL) for 7 days. Raw 264.7 cell lines (RRID: CVCL_0493) and HEK293T cell lines (RRID: CVCL_0063) were purchased from Zhejiang Meisen Cell Technology (CTCC‐001‐0048 and CTCC‐001‐0188). Both cell lines were routinely tested for mycoplasma contamination using PCR Mycoplasma Test Kit (HUABIO, K0103) and results confirmed no contamination. All small‐molecule compounds used in all cell treatments are listed in Table S2. Cell viability following all pharmacological treatments was assessed using the CCK‐8 assay, which confirmed no significant cytotoxic effect of any treatment.

### 4.7 Gene Silencing and Overexpression

Small interfering RNA (siRNA) targeting F13A1, designed and synthesized by Hanbio Biotechnology (Shanghai, China) with sequences matching adeno‐associated virus constructs, was delivered via lipid nanoparticles (LNP) to primary macrophages [59, 60]. A non‐targeting scrambled siRNA served as a negative control. Knockdown efficiency was validated by qRT‐PCR. For overexpression, plasmids encoding Flag‐tagged F13A1 and HA‐tagged PKM2, designed and produced by Transheep Biotech (Shanghai, China), were transfected into RAW264.7 and HEK293T cells using Lipofect 5000 (Bio‐generating, 21051). Overexpression was confirmed by Western blot detecting Flag and HA tags.

### 4.8 Transcriptomics, Proteomics, and Lipidomics

For transcriptomic analysis, total RNA was extracted from BMDMs and subjected to high‐throughput sequencing (RNA‐seq) on an Illumina NovaSeq 6000 platform. Service provided by LC‐Bio Technologies (Hangzhou, China). Proteomic profiling was conducted using liquid chromatography‐tandem mass spectrometry (LC‐MS/MS), also provided by LC‐Bio Technologies. Lipidomic analysis was conducted by Hangzhou Cosmos WisdomBiotech (Hangzhou, China). For transcriptomics data, differential gene expression analysis was performed using the moderated *t*‐test in the limma package (v3.56.2), with *p*‐values adjusted using the Benjamini–Hochberg (BH) method to control the False Discovery Rate (FDR). For metabolomics data, differential metabolite analysis was conducted using the Student's *t*‐test, with *p*‐values adjusted using the Benjamini‐Hochberg (BH) method to control the False Discovery Rate (FDR).

### 4.9 Histological and Immunostaining Procedures

Formalin‐fixed, paraffin‐embedded liver sections (4 µm) were stained with hematoxylin‐eosin (HE) for histopathologic evaluation and Masson's trichrome for fibrosis. Oil Red O staining was performed on frozen sections to assess hepatic lipid deposition. Apoptotic cells were detected using TUNEL assay (Roche). Immunohistochemistry (IHC) was performed using antibodies against F13A1, MPO, F4/80, and LY6G, followed by HRP‐conjugated secondary antibodies and DAB development. Multiplex immunofluorescence staining was performed using a tyramide signal amplification (TSA) system according to the manufacturer's instructions. Slides were imaged with a confocal microscope, and quantitative analyses were conducted using ImageJ.

### 4.10 PCR, Western Blot, and Flow Cytometry

Quantitative real‐time PCR (qRT‐PCR) was performed using Universal SYBR Green Fast qPCR Mix (Abclonal Technology, RK21203). RNA was extracted with AFTSpin Tissue/Cell Fast RNA Extraction Kit (Abclonal Technology, RK30120), reverse‐transcribed with ABScript Neo RT Master Mix for qPCR (Abclonal Technology, RK20433), and amplified using specific primers. Relative gene expression was quantified using the 2^‐ΔΔCt method, with ACTB as the reference gene. Western blot (WB) analysis was conducted using standard protocols, with proteins separated by SDS‐PAGE, transferred to PVDF membranes, and probed with specific antibodies. Bands were quantified using ImageJ by measuring integrated density after background subtraction. Target protein levels were normalized to loading control, then expressed as fold change relative to control group. For flow cytometry, cells were stained with fluorochrome‐conjugated antibodies, fixed, and analyzed for surface marker expression. Data were processed and quantified using FlowJo (v10.8.1). All antibodies used are listed in Table S3, and all primers are listed in Table S4.

### 4.11 Co‐Immunoprecipitation and Chromatin Immunoprecipitation

Co‐immunoprecipitation (Co‐IP) was performed using Protein A/G magnetic beads (Selleck, B23201) incubated with primary antibodies, or using Anti‐Flag magnetic bead (Selleck, B26101), Anti‐HA magnetic beads (Selleck, B26201), incubated with cell lysates overnight at 4°C, followed by bead capture, washing, and elution for WB analysis. Chromatin Immunoprecipitation (ChIP) assays were performed using the Abclonal ChIP kit (Abclonal Technology, RK20258), following the manufacturer's instructions. Primers for ChIP‐PCR are listed in Table S5.

### 4.12 Glycolytic Function Assay

Glycolytic function was assessed using a Seahorse XFe96 Analyzer (Agilent) with the Glycolysis Stress Test Kit. Cells (2×10⁴/well) were incubated in glucose‐free XF Base Medium + 2 mm glutamine for 1 h (37 °C, non‐CO_2_), then sequentially injected with 10 mm glucose, 1 µm oligomycin, and 50 mm 2‐DG while measuring ECAR. Glycolysis rate = ECAR after glucose injection—basal ECAR; glycolytic capacity = ECAR after oligomycin—basal ECAR. Intracellular succinate was detected based on the AB Sciex OTRAP 6500 LC‐MS/MS platform and was conducted by Hangzhou Cosmos WisdomBiotech (Hangzhou, China).

### 4.13 Statistical Analysis

Statistical analyses were performed using R (version 4.3.1) and GraphPad Prism (version 9.5). For Western blot and qPCR data, protein or mRNA expression levels were normalized to the corresponding control group. Normality was assessed using the Shapiro‐Wilk test. Data are presented as mean ± SD or median with interquartile range according to distribution. Sample sizes (n) are indicated in the figure legends. All statistical tests were two‐sided, and significance was defined as *p* < 0.05.

Comparisons between two groups were performed using the unpaired Student's *t*‐test (parametric) or Mann–Whitney U test (non‐parametric). Multiple group comparisons were analyzed by one‐way ANOVA followed by Dunnett's post hoc test (each group vs. control), or by two‐way ANOVA followed by Tukey's post hoc test. When data were not normally distributed, the Kruskal–Wallis test followed by Dunn's post hoc test with Bonferroni correction was used. Associations between gene expression and NAS were evaluated using univariate logistic regression.

## Conflicts of Interest

The authors declare no conflict of interest.

## Supporting information




**Supporting File**: advs73415‐sup‐0001‐SuppMat.pdf.

## Data Availability

The data that support the findings of this study are available from the corresponding author upon reasonable request.
