# Supplementary material for: F13A1‐Mediated Macrophage Activation Promotes MASH Progression via the PKM2/HIF1A Pathway
Source: Adv Sci (Weinh). 2025 Dec 19;13(13):e18128. doi: 10.1002/advs.202518128 (PMC12955996; doi:10.1002/advs.202518128)
Supplement: Supplementary file 1 — Supporting File: advs73415‐sup‐0001‐SuppMat.pdf. [file ADVS-13-e18128-s001.pdf]

F13A1-Mediated Macrophage Activation Promotes MASH Progression via the PKM2/HIF1A Pathway

Qianrang Lu, Meiching Ong, Xuewen Yi, Muqiong Xing, Xinyao Tian, Ke Zhang, Ling Lu, Hao Wang, Xiaohan Lin, Jun Fang, Aibo Mu, Jiaying Cao, Jingyu Jiang, Feng Gao, Hongjun Li, Baohong Wang, Qi Ling *

Supplemental Figure 1

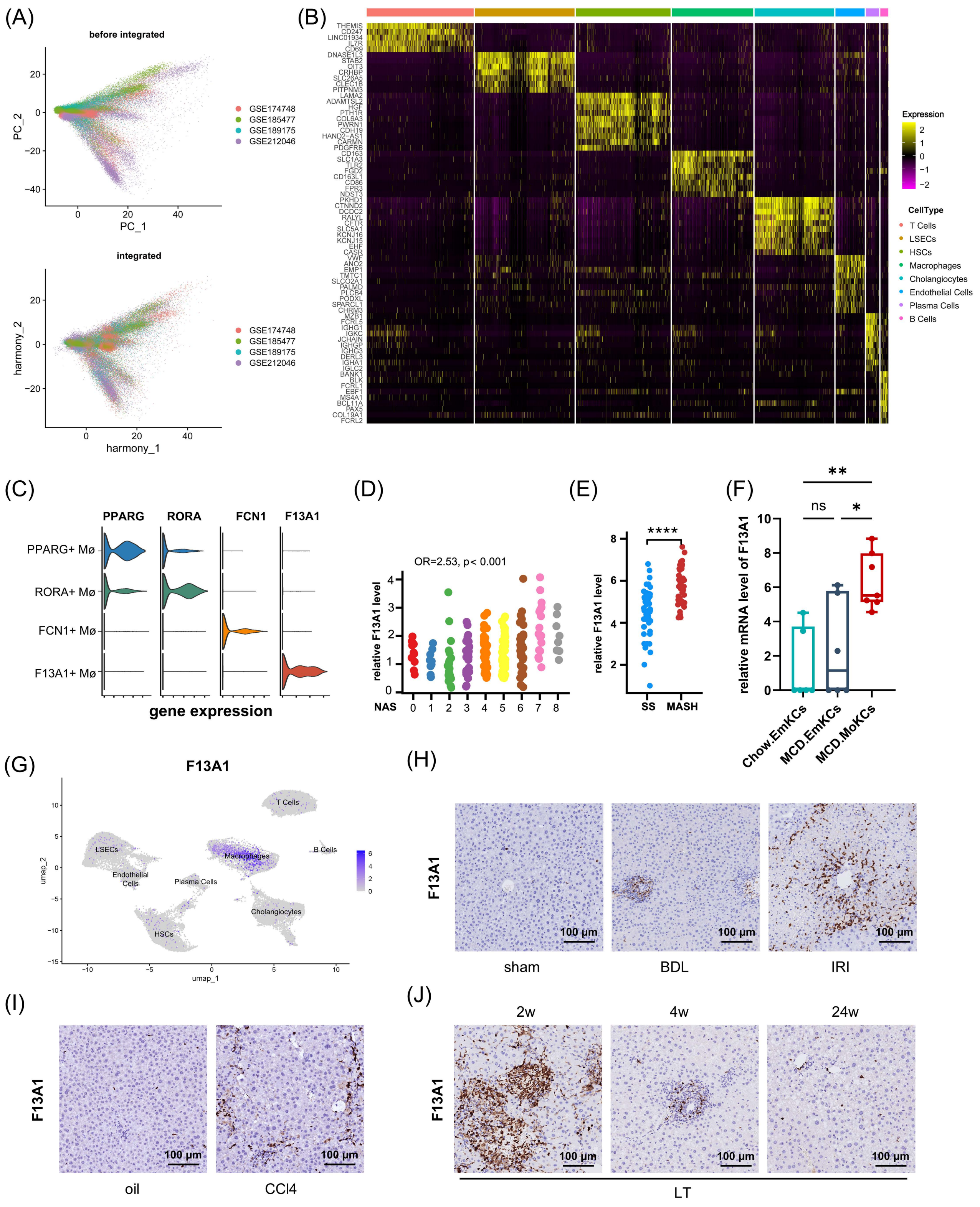

Figure S1. (A) Integration of four GSE datasets (GSE174748, GSE185477, GSE189175, GSE212046); PCA plots before and after integration. (B) Heatmap of the top 10 marker genes for each NPC cluster. (C) Violin plots of representative marker genes for each macrophage cluster. (D) Correlation between hepatic *F13A1* expression and NAS in GSE135251. (E) Hepatic *F13A1* expression in simple steatosis (SS) vs. MASH in GSE167523. (F) *F13a1* expression in embryonic Kupffer cells from CD-fed mice, embryonic Kupffer cells from MCD-fed mice, and monocyte-derived Kupffer cells from MCD-fed mice (GSE138778). (G) Expression of F13A1 in all hepatic NPC clusters. (H–J)

Immunohistochemistry of F13A1 expression in BDL, IRI, CCl_4 mouse models and mouse liver transplantation at 2, 4, and 24 weeks. Data are presented as median with interquartile range in (F). For (D), significance was determined by univariate logistic regression. For (E), significance was determined by Student's t-test. For (F), significance was determined by the Kruskal-Wallis test. ns = no significance, *$P < 0.05$, **$P < 0.01$, ***$P < 0.001$, ****$P < 0.0001$. Abbreviations: F13A1, Coagulation Factor XIII Subunit A; PCA, Principal Component Analysis; NPC, Non-Parenchymal Cell; NAS, non-alcoholic steatohepatitis; MASH, metabolic-associated steatohepatitis; CD, Chow-diet; MCD, Methionine–Choline Deficient diet.

Supplemental Figure 2

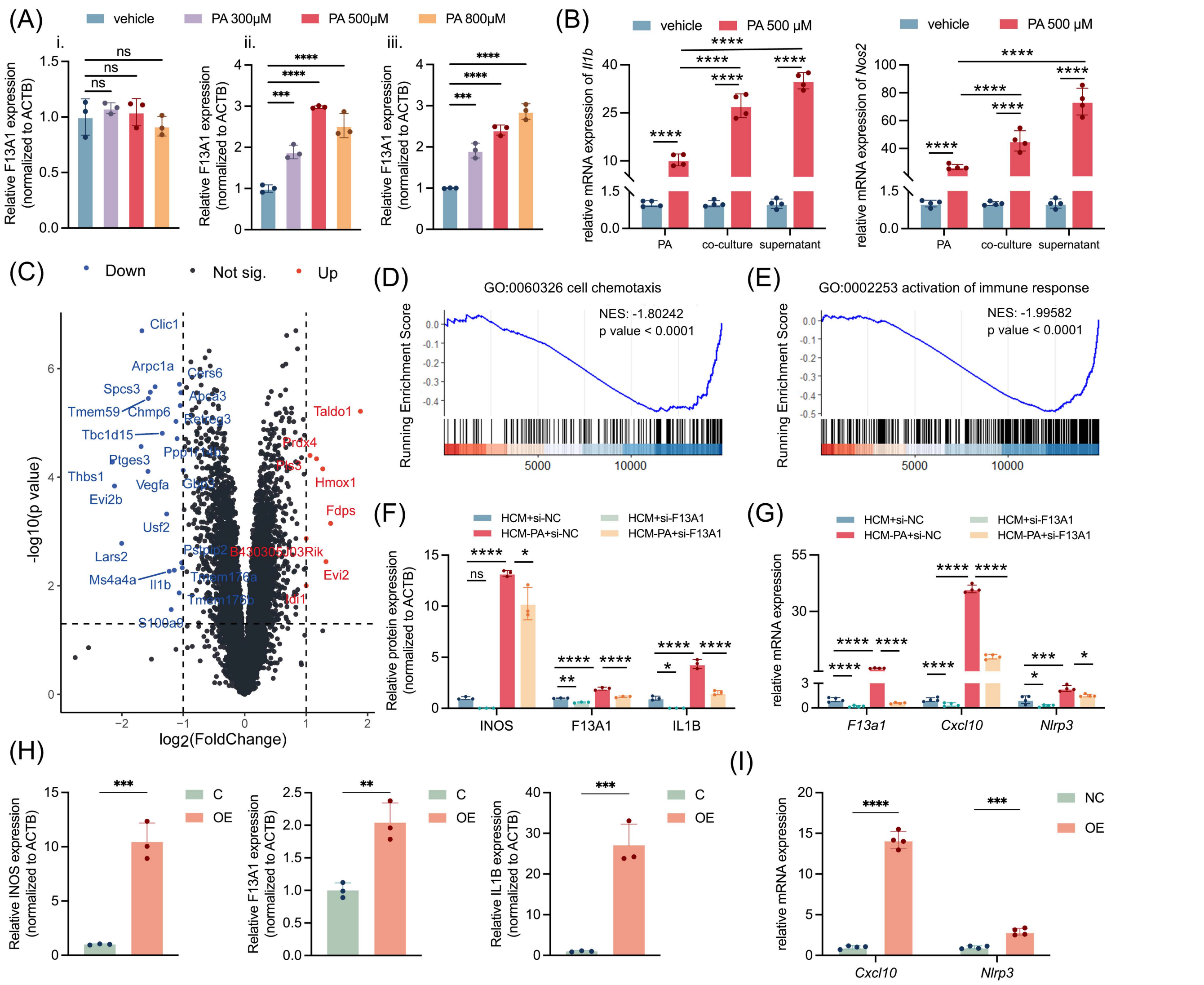

Figure S2. (A) Relative quantification of Western blot in Figure 2C. (B) BMDMs stimulated for 24 h with palmitic acid (500 μM) direct, hepatocyte co-culture, or hepatocyte-conditioned medium (HCM-PA/HCM); mRNA levels of *Il1b* and *Nos2* detected. (C) RNA-seq of BMDMs after F13A1 knockdown (24 h); volcano plot of differentially expressed genes ($p<0.05$, |log2FoldChange|>1). (D, E) GSEA showing reduced chemotaxis and immune activation pathways after F13A1 knockdown. (F) Relative quantification of Western blot in Figure 2F. (G) BMDMs transfected with si-F13A1 or si-NC (12 h), then treated with HCM or HCM-PA (24 h); qPCR of *F13a1*, *Cxcl10, Nlrp3* mRNA. (H) Relative quantification of Western blot in Figure 2I. (I) RAW264.7 cells transfected with F13A1 plasmid (24 h); qPCR of *Cxcl10* and *Nlrp3* mRNA. Data are presented as mean ± standard deviation. For (A), significance was determined by one-way ANOVA. For (F, G), significance was determined by two-way ANOVA. For (B, H, I), significance was determined by Student's t-test. ns = no significance, *P < 0.05, **P < 0.01, ***P < 0.001, ****P < 0.0001. Abbreviations: F13A1, Coagulation Factor XIII Subunit A; BMDM, Bone Marrow-Derived Macrophage; PA, palmitic acid; HCM, Hepatocyte-Conditioned Medium; HCM-PA, Palmitic Acid-Treated Hepatocyte-Conditioned Medium; siRNA, Small Interfering RNA; NC, negative control; CXCL10, Chemokine (C-X-C motif) Ligand 10; NLRP3, NACHT, LRR and PYD domains-containing protein 3.

Supplemental Figure 3

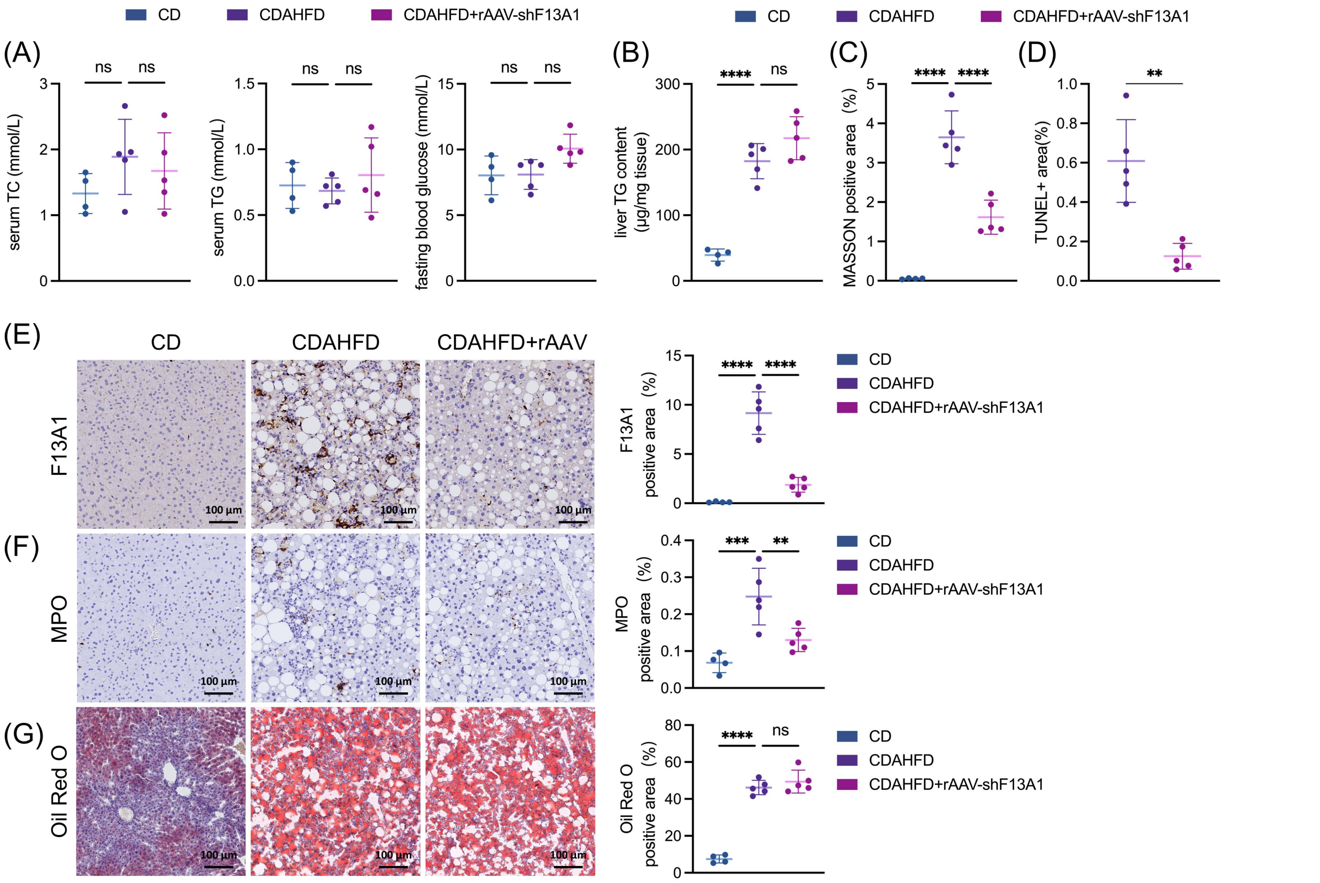

Figure S3. (A) Serum TC, TG, and fasting glucose after 3 weeks of rAAV treatment. (B) Hepatic TG content after 3 weeks of rAAV treatment. (C, D) Quantification of positive staining areas for Masson and TUNEL. (E, F) Immunohistochemistry of F13A1 and MPO in each group, and quantification of positive staining areas. (G) Oil Red O staining in each group, and quantification of positive staining areas. Data are presented as mean ± standard deviation. For (A-C) and (E-G) significance was determined by one-way ANOVA. For (D), significance was determined by Student's t-test. ns = no significance, *P < 0.05, **P < 0.01, ***P < 0.001, ****P < 0.0001. Abbreviations: MASH, Metabolic-Associated Steatohepatitis; rAAV, Recombinant Adeno-Associated Virus; TC, Total Cholesterol; TG, triglyceride; MPO, Myeloperoxidase.

Supplemental Figure 4

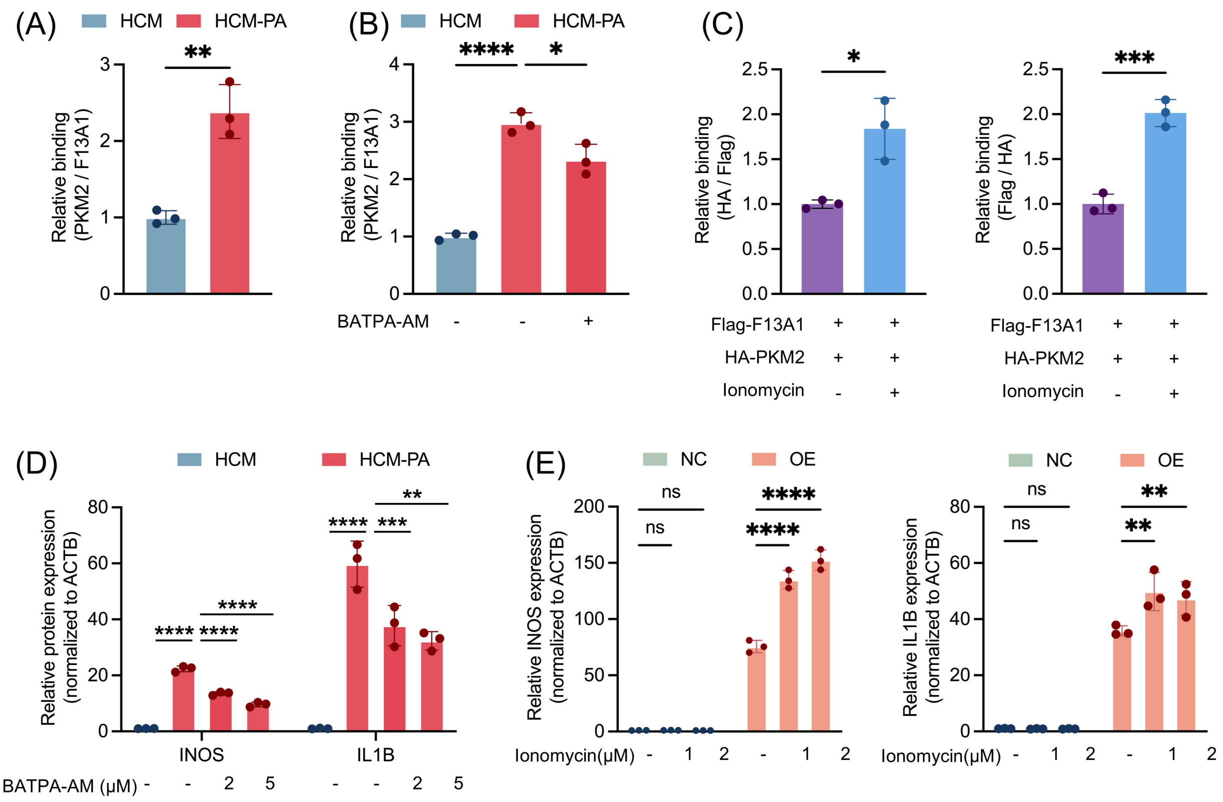

Figure S4. (A) Relative quantification of Western blot in Figure 4C. (B) Relative quantification of Western blot in Figure 4E. (C) Relative quantification of Western blot in Figure 4F. (D) Relative quantification of Western blot in Figure 4H. (E) Relative quantification of Western blot in Figure 4I. Data are presented as mean ± standard deviation. For (A, C), significance was determined by Student's t-test. For (B, D), significance was determined by one-way ANOVA. For (E), significance was determined by two-way ANOVA. ns = no significance, *P < 0.05, **P < 0.01, ***P < 0.001, ****P < 0.0001.

Supplemental Figure 5

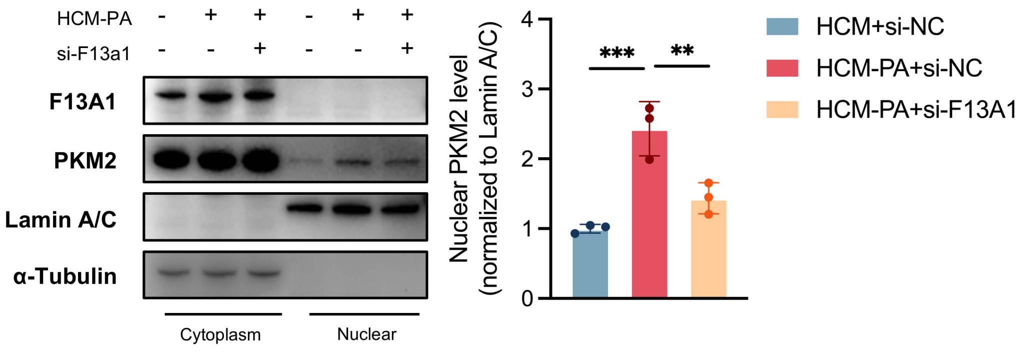

Figure S5. BMDMs transfected with si-F13A1 or si-NC (12 h), then treated with HCM-PA or HCM(12 h); Western blot of F13A1 expression and cytoplasmic/nuclear PKM2 distribution, along with relative quantification of nuclear PKM2. Data are presented as mean ± standard deviation. Significance was determined by one-way ANOVA. **P < 0.01, ***P < 0.001.

Supplemental Figure 6

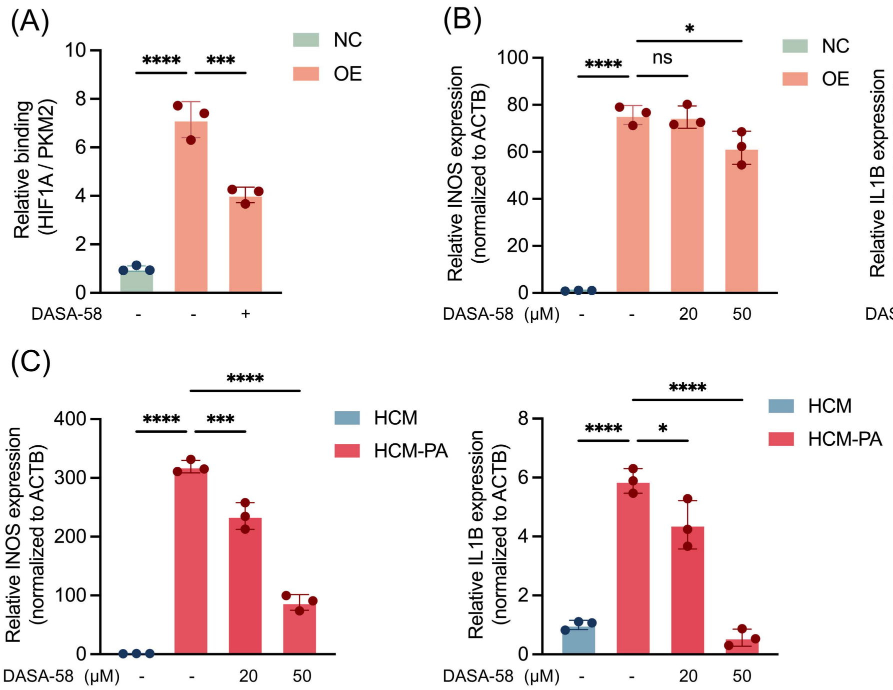

Figure S6. (A) Relative quantification of Western blot in Figure 6A. (B) Relative quantification of Western blot in Figure 6G. (C) Relative quantification of Western blot in Figure 6H. Data are presented as mean ± standard deviation. Significance was determined by one-way ANOVA. ns = no significance, *P < 0.05, **P < 0.01, ***P < 0.001, ****P < 0.0001.

Supplemental Figure 7

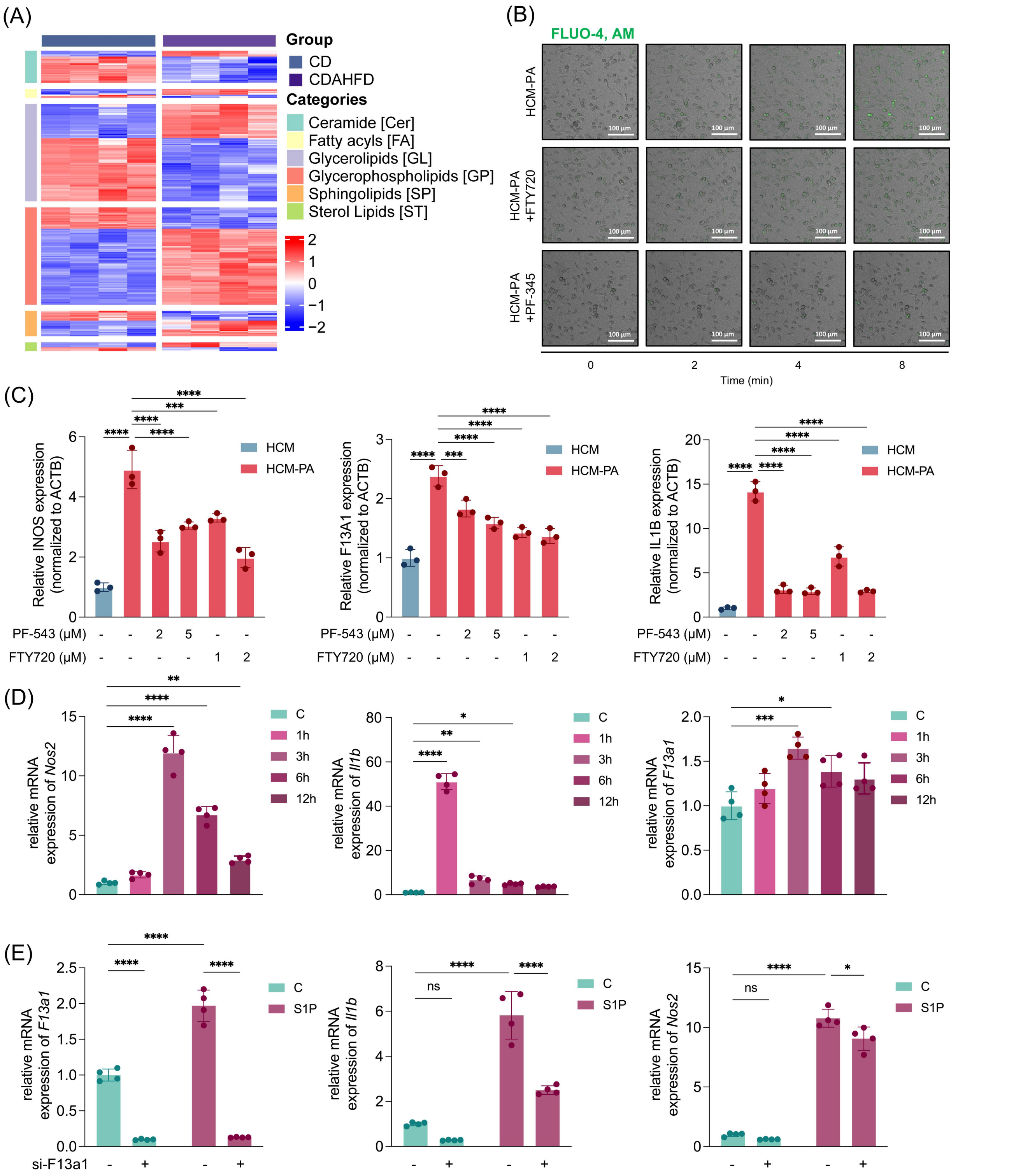

Figure S7. (A) Differential hepatic lipid species between CDAHFD-fed mice (8 weeks) and CD controls (FDR<0.05); top 100 compounds per lipid class ranked by FDR. (B) Hepatocytes treated with palmitic acid (500 μM, 24 h) to generate HCM-PA, with or without PF-543 (5 μM) to inhibit S1P synthesis; or HCM-PA used to stimulate BMDMs with/without FTY720 pretreatment (2 μM, 2 h) to block S1P function; confocal microscopy showing representative fields of intracellular fluorescence intensity in BMDMs. (C) Relative quantification of Western blot in Figure 7D. (D) BMDMs stimulated with S1P (2 μM) for 1, 2, 3, 6, or 12 h; qPCR of *F13a1, Il1b,* and *Nos2* mRNA. (E) BMDMs transfected with si-F13A1 or si-NC (12 h), then stimulated with S1P (2 μM, 3 h); qPCR of *F13a1, Il1b,* and *Nos2* mRNA. Data are presented as mean ± standard deviation. For (C, D) significance was determined by one-way ANOVA. For (E) significance was determined by two-way ANOVA. ns = no significance, *P < 0.05, **P < 0.01, ***P <

0.001, ****P < 0.0001. Abbreviations: MASH, Metabolic-Associated Steatohepatitis; BMDM, Bone Marrow-Derived Macrophage; F13A1, Coagulation Factor XIII Subunit A; CDAHFD, Choline-Deficient, L-Amino Acid-Defined, High-Fat Diet; CD, Chow-diet; FDR, False Discovery Rate; HCM-PA, Palmitic Acid-Treated Hepatocyte-Conditioned Medium; S1P, Sphingosine-1-Phosphate.

Supplemental Figure 8

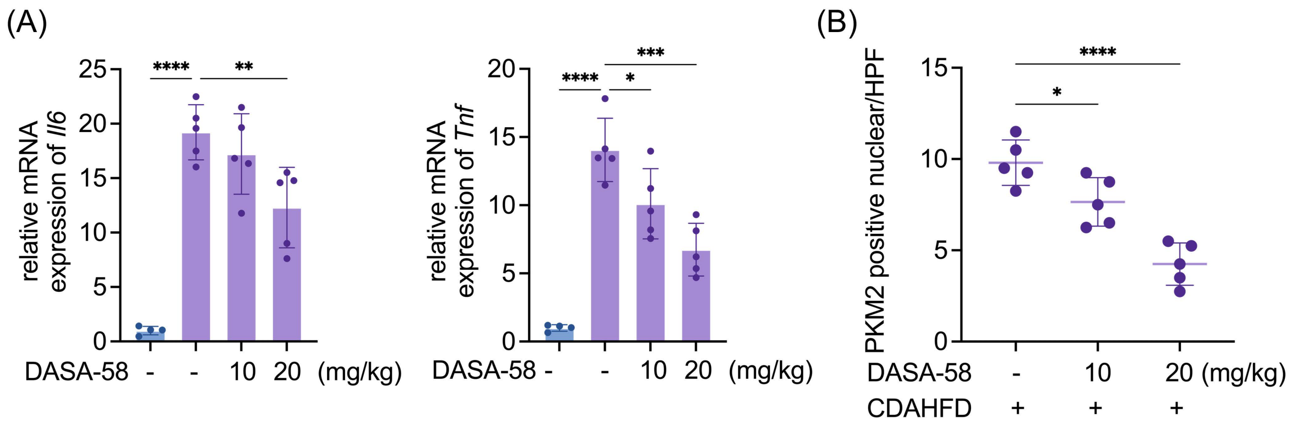

Figure S8. (A) Primary hepatic macrophages isolated from each group; qPCR of *Il6* and *Tnf*. (B) Immunofluorescence of PKM2 and F13A1 in liver from CDAHFD and treated mice; quantification of PKM2-positive nuclei per HPF. Data are presented as mean ± standard deviation. Significance was determined by one-way ANOVA. ns = no significance, *P < 0.05, **P < 0.01, ***P < 0.001, ****P < 0.0001.

Table S1. Gene markers of each macrophage clusters, p_val < 0.05, avg_log2FC > 1

gene symbol	p_val	avg_log2FC	pct.1	pct.2	p_val_adj	cluster
EXT1	3.681E-167	1.9214537	0.933	0.619	9.7244E-163	PPARG+ Mø
CCDC26	2.843E-163	1.9633678	0.648	0.173	7.5126E-159	PPARG+ Mø
KIAA1217	4.482E-163	1.8516314	0.686	0.189	1.1843E-158	PPARG+ Mø
LILRB5	1.582E-114	1.197973	0.651	0.212	4.18E-110	PPARG+ Mø
TMEM26	1.038E-106	1.3576646	0.417	0.088	2.7415E-102	PPARG+ Mø
NDST3	3.11E-105	1.0669794	0.667	0.218	8.2168E-101	PPARG+ Mø
UNC5C	8.58E-104	1.5733803	0.375	0.07	2.267E-99	PPARG+ Mø
DSCAM	1.001E-101	1.4169948	0.552	0.169	2.64393E-97	PPARG+ Mø
TPRG1	1.1573E-94	1.2206626	0.743	0.331	3.05778E-90	PPARG+ Mø
FRMD4B	5.0203E-93	1.0447098	0.895	0.617	1.32642E-88	PPARG+ Mø
ANKRD36C	7.1001E-88	1.5096333	0.606	0.245	1.87592E-83	PPARG+ Mø
MYO9A	2.806E-86	1.0834828	0.755	0.405	7.41379E-82	PPARG+ Mø
PPARG	2.4188E-84	1.33734	0.581	0.234	6.39081E-80	PPARG+ Mø
ME1	9.7077E-84	1.1045031	0.688	0.321	2.56488E-79	PPARG+ Mø
RGL1	2.4019E-83	1.0358372	0.844	0.516	6.346E-79	PPARG+ Mø
PDE4DIP	3.7263E-81	1.2322724	0.613	0.271	9.84521E-77	PPARG+ Mø
ANKH	2.584E-80	1.5286768	0.697	0.368	6.82721E-76	PPARG+ Mø
TIMD4	5.2614E-79	1.1123889	0.257	0.038	1.39011E-74	PPARG+ Mø
MPPED2	1.9949E-78	1.312556	0.514	0.184	5.27063E-74	PPARG+ Mø
ABCA1	7.3898E-77	1.081166	0.808	0.545	1.95245E-72	PPARG+ Mø
WSB1	4.4441E-75	1.0675776	0.834	0.581	1.17416E-70	PPARG+ Mø
VCAM1	7.8327E-74	1.2041147	0.448	0.144	2.06948E-69	PPARG+ Mø
FARP1	1.5072E-73	1.1012922	0.559	0.226	3.98211E-69	PPARG+ Mø
ROR1	9.9619E-68	1.1189279	0.281	0.06	2.63202E-63	PPARG+ Mø
HMOX1	1.404E-64	1.1679855	0.44	0.16	3.7094E-60	PPARG+ Mø
CD5L	9.7049E-58	1.0110944	0.304	0.083	2.56412E-53	PPARG+ Mø
IPCEF1	1.9349E-55	1.1238154	0.341	0.108	5.11214E-51	PPARG+ Mø
FGF13	4.6381E-45	1.1510147	0.32	0.111	1.22543E-40	PPARG+ Mø
CCL3	7.9779E-38	1.0094848	0.255	0.084	2.10783E-33	PPARG+ Mø
MMP19	5.7139E-37	1.1351971	0.255	0.086	1.50968E-32	PPARG+ Mø
F13A1	4.098E-179	4.0398183	0.515	0.084	1.0828E-174	F13A1+ Mø
SH3PXD2B	3.883E-169	2.0948462	0.665	0.188	1.026E-164	F13A1+ Mø
FMN1	2.845E-152	2.0947957	0.832	0.467	7.5171E-148	F13A1+ Mø
CSGALNACT1	4.693E-152	2.3431673	0.557	0.133	1.2399E-147	F13A1+ Mø
USP53	1.301E-136	2.1461392	0.448	0.082	3.4367E-132	F13A1+ Mø
PALD1	3.534E-109	1.5693189	0.393	0.075	9.3379E-105	F13A1+ Mø
SLC1A3	9.795E-100	1.5206141	0.77	0.424	2.58787E-95	F13A1+ Mø
IGSF21	1.8645E-98	2.19549	0.417	0.108	4.9262E-94	F13A1+ Mø
SRGAP1	9.9836E-95	1.9393265	0.633	0.317	2.63778E-90	F13A1+ Mø
ADAM28	2.017E-93	1.504478	0.363	0.074	5.32899E-89	F13A1+ Mø
ZBTB16	2.3609E-92	1.5517007	0.665	0.303	6.23782E-88	F13A1+ Mø
CPM	3.8543E-90	1.5949415	0.685	0.356	1.01833E-85	F13A1+ Mø
FKBP5	7.8411E-85	1.1185498	0.864	0.673	2.07169E-80	F13A1+ Mø
STAB1	1.2846E-82	1.2560812	0.513	0.182	3.39403E-78	F13A1+ Mø
RNASET2	1.8422E-77	1.4125053	0.422	0.13	4.86723E-73	F13A1+ Mø
APOC2	1.305E-76	1.2451004	0.337	0.078	3.44783E-72	F13A1+ Mø
FAM53B	1.5198E-73	1.3112276	0.339	0.086	4.01535E-69	F13A1+ Mø
PLXDC2	1.0117E-71	1.2028467	0.824	0.642	2.67291E-67	F13A1+ Mø
MS4A7	1.0625E-68	1.2077466	0.408	0.131	2.80714E-64	F13A1+ Mø
MGAT4A	1.2073E-66	1.3889351	0.435	0.161	3.18982E-62	F13A1+ Mø
SLCO2B1	7.9151E-65	1.3927066	0.691	0.451	2.09126E-60	F13A1+ Mø
CD74	1.3931E-63	1.1440674	0.649	0.364	3.68072E-59	F13A1+ Mø
BNC2	6.5574E-63	1.5355728	0.299	0.076	1.73254E-58	F13A1+ Mø
MS4A4A	2.155E-62	1.3544173	0.472	0.202	5.69366E-58	F13A1+ Mø
DTNA	1.0162E-61	1.4904887	0.317	0.086	2.68479E-57	F13A1+ Mø

CHST11	1.0009E-59	1.2470442	0.714	0.474	2.64437E-55	F13A1+ Mø
MS4A4E	1.003E-55	1.2238827	0.58	0.315	2.65009E-51	F13A1+ Mø
PPP3R1	1.1695E-54	1.2755082	0.298	0.088	3.08992E-50	F13A1+ Mø
TRPM2	1.559E-53	1.1397987	0.353	0.122	4.11905E-49	F13A1+ Mø
SFMBT2	3.5815E-53	1.5234891	0.541	0.287	9.46276E-49	F13A1+ Mø
SRGAP2	2.3537E-52	1.1317136	0.638	0.416	6.21861E-48	F13A1+ Mø
ELMO1	9.0845E-52	1.0503343	0.768	0.659	2.40022E-47	F13A1+ Mø
SRGAP2B	4.8673E-51	1.2081298	0.567	0.335	1.28599E-46	F13A1+ Mø
DAB2	2.8002E-50	1.1570212	0.28	0.083	7.39829E-46	F13A1+ Mø
HLA-DRB5	7.2423E-49	1.0152574	0.348	0.13	1.91349E-44	F13A1+ Mø
ARHGAP18	2.0005E-47	1.2927148	0.574	0.343	5.28546E-43	F13A1+ Mø
AHR	3.5115E-45	1.1576584	0.356	0.144	9.27773E-41	F13A1+ Mø
PLTP	1.9031E-44	1.0323218	0.317	0.112	5.0282E-40	F13A1+ Mø
PCNX1	4.1756E-44	1.0352626	0.487	0.242	1.10323E-39	F13A1+ Mø
CHPT1	9.8074E-44	1.014126	0.381	0.162	2.59122E-39	F13A1+ Mø
GALNT2	1.2897E-43	1.0633101	0.469	0.235	3.4076E-39	F13A1+ Mø
FCGR2A	3.974E-43	1.0256888	0.402	0.183	1.04997E-38	F13A1+ Mø
TANC2	7.899E-41	1.1309143	0.477	0.253	2.087E-36	F13A1+ Mø
RTN1	2.1246E-40	1.0045915	0.386	0.167	5.61334E-36	F13A1+ Mø
PREX1	2.2828E-39	1.0206185	0.585	0.385	6.03142E-35	F13A1+ Mø
TBC1D14	1.7714E-38	1.0612429	0.494	0.284	4.68026E-34	F13A1+ Mø
PRKCE	2.556E-36	1.007199	0.592	0.376	6.75313E-32	F13A1+ Mø
C5orf17	8.7219E-35	1.035443	0.286	0.108	2.30441E-30	F13A1+ Mø
RGS1	1.4891E-33	1.2207549	0.319	0.14	3.93448E-29	F13A1+ Mø
ARHGAP22	3.2821E-33	1.0012509	0.264	0.099	8.67158E-29	F13A1+ Mø
MAN1A1	5.3978E-29	1.0568762	0.631	0.462	1.42614E-24	F13A1+ Mø
DSE	1.5042E-28	1.0021845	0.369	0.194	3.97429E-24	F13A1+ Mø
MGAT5	5.5408E-27	1.1586887	0.54	0.372	1.46393E-22	F13A1+ Mø
CD163L1	6.2118E-17	1.3215499	0.506	0.378	1.64121E-12	F13A1+ Mø
NHSL1	1.3635E-15	1.0900692	0.501	0.385	3.60251E-11	F13A1+ Mø
VCAN	8.773E-169	3.3516376	0.482	0.069	2.318E-164	FCN1+ Mø
FCN1	7.158E-113	1.7672167	0.282	0.025	1.8913E-108	FCN1+ Mø
PLXNC1	2.713E-70	1.295654	0.378	0.106	7.16801E-66	FCN1+ Mø
EMILIN2	8.0397E-57	1.2217911	0.407	0.148	2.12417E-52	FCN1+ Mø
MAP4K4	1.3253E-56	1.240316	0.356	0.111	3.50152E-52	FCN1+ Mø
SLC11A1	5.5532E-56	1.7156193	0.468	0.201	1.46722E-51	FCN1+ Mø
XYLT1	1.4873E-54	1.1322174	0.438	0.17	3.92962E-50	FCN1+ Mø
ITGAX	2.7643E-54	1.4542158	0.494	0.223	7.30351E-50	FCN1+ Mø
FYN	5.5673E-54	1.3045294	0.296	0.081	1.47093E-49	FCN1+ Mø
JAML	2.8574E-50	1.1807903	0.326	0.106	7.54958E-46	FCN1+ Mø
LYST	2.8307E-43	1.3416705	0.574	0.358	7.47908E-39	FCN1+ Mø
LYZ	3.2563E-35	1.3761673	0.27	0.097	8.60344E-31	FCN1+ Mø
RAP1GAP2	3.6688E-35	1.1438246	0.284	0.107	9.69326E-31	FCN1+ Mø
ATP13A3	8.3023E-34	1.1810791	0.483	0.277	2.19354E-29	FCN1+ Mø
CREB5	1.6248E-33	1.0387941	0.307	0.123	4.29282E-29	FCN1+ Mø
PLCB1	5.1652E-27	1.4385977	0.363	0.19	1.36469E-22	FCN1+ Mø
LRRK2	5.5323E-27	1.0479888	0.492	0.307	1.4617E-22	FCN1+ Mø
ASPH	3.9378E-24	1.1846892	0.327	0.167	1.0404E-19	FCN1+ Mø
MTRNR2L12	4.9747E-23	1.6073418	0.274	0.134	1.31437E-18	FCN1+ Mø
MEGF9	5.1336E-18	1.0288264	0.366	0.227	1.35634E-13	FCN1+ Mø
SAA1	3.5608E-14	1.5127036	0.325	0.209	9.408E-10	FCN1+ Mø
TFRC	1.4098E-13	1.1520866	0.256	0.147	3.7247E-09	FCN1+ Mø
C10orf11	1.605E-204	2.2845874	0.598	0.096	4.2397E-200	RORA+ Mø
FYB	4.695E-141	1.8277611	0.463	0.078	1.2406E-136	RORA+ Mø
RP11-1080G15.1	4.123E-128	1.7439891	0.284	0.015	1.0893E-123	RORA+ Mø
PARK2	1.785E-117	1.6469052	0.294	0.025	4.7156E-113	RORA+ Mø
GMDS-AS1	1.209E-112	1.6579843	0.334	0.045	3.1932E-108	RORA+ Mø
LINC-PINT	7.754E-109	1.8790615	0.462	0.129	2.0486E-104	RORA+ Mø

RP11-426C22.5	3.56E-107	1.5315472	0.356	0.058	9.4051E-103	RORA+ Mø
RFWD2	1.24E-104	1.374916	0.388	0.073	3.2772E-100	RORA+ Mø
LINC00486	5.668E-99	1.640955	0.34	0.061	1.49754E-94	RORA+ Mø
TMEM2	2.3361E-98	1.5038621	0.311	0.046	6.17217E-94	RORA+ Mø
CPS1	2.4445E-92	1.4694063	0.621	0.284	6.45855E-88	RORA+ Mø
ZCCHC6	4.3068E-89	1.3469838	0.338	0.066	1.13791E-84	RORA+ Mø
BRE	5.4241E-85	1.2058636	0.271	0.039	1.43311E-80	RORA+ Mø
PAPD4	2.7887E-84	1.3045236	0.287	0.048	7.36798E-80	RORA+ Mø
RORA	9.9355E-83	1.4191326	0.513	0.201	2.62505E-78	RORA+ Mø
CYP2B6	1.1196E-77	1.6459719	0.459	0.171	2.95806E-73	RORA+ Mø
CYP2C8	5.614E-69	1.228073	0.552	0.254	1.48327E-64	RORA+ Mø
SORBS2	3.6165E-63	1.2775469	0.38	0.129	9.55526E-59	RORA+ Mø
NDST3	1.3091E-62	1.3425615	0.526	0.249	3.4587E-58	RORA+ Mø
CHN2	4.9447E-61	1.2568222	0.336	0.107	1.30643E-56	RORA+ Mø
GPC6	8.8832E-60	1.198486	0.415	0.159	2.34702E-55	RORA+ Mø
PPARGC1A	3.09E-59	1.3539628	0.292	0.08	8.16409E-55	RORA+ Mø
TSHZ2	6.1189E-59	1.3264286	0.324	0.099	1.61669E-54	RORA+ Mø
CYP3A5	3.1478E-57	1.1066571	0.422	0.165	8.31676E-53	RORA+ Mø
MYO1B	1.7487E-47	1.1618426	0.28	0.089	4.62019E-43	RORA+ Mø
MAGI1	6.083E-44	1.1725488	0.345	0.141	1.60718E-39	RORA+ Mø
MARCO	2.9656E-36	1.2975568	0.412	0.215	7.83528E-32	RORA+ Mø
PCK1	7.0083E-36	1.0670419	0.387	0.194	1.85166E-31	RORA+ Mø
HGD	1.9004E-35	1.0879954	0.263	0.1	5.02108E-31	RORA+ Mø
ERRFI1	4.0677E-34	1.0330542	0.351	0.171	1.07474E-29	RORA+ Mø
AP2A2	3.6202E-30	1.0341989	0.65	0.526	9.56486E-26	RORA+ Mø
PAH	1.6033E-29	1.009427	0.278	0.122	4.2361E-25	RORA+ Mø

Supplemental Table S2. Small Molecule Compounds Used in This Study

Chemical name	Supplier	Catalog number
DASA-58	MCE	HY-19330
BATPA-AM	MCE	HY-100545
Ionomycin	MCE	HY-13434
FTY720	MCE	HY-12005
PF-543	MCE	HY-15425
Sphingosine 1-phosphate	Merck	SML2709
lipopolysaccharide	Merck	L8274
palmitic acid	aladdin	P432957

Supplemental Table S3. Antibodies Used in This Study

Antibody	Host species	Application	Supplier	Catalog number
F13A1	Rabbit	WB	ABclonal	A1461
PKM2	Rabbit	WB, Co-IP, ICC	ABclonal	A20991
IL1B	Rabbit	WB	ABclonal	A27676
iNOS	Rabbit	WB	ABclonal	A3774
ACTB	Rabbit	WB	ABclonal	AC026
α-Tubulin	Rabbit	WB	ABclonal	A6830
Lamin B	Rabbit	WB	ABclonal	A11495
HIF1α	Rabbit	WB	ABclonal	A26889
NF-kB	Rabbit	WB	ABclonal	A19653
p-NF-kB	Rabbit	WB	ABclonal	AP1294
F13A1	Rabbit	IHC, IF, Co-IP	abcam	ab76105
F13A1	Mouse	WB	abcam	ab1834
PKM2	Mouse	WB, IF	proteintech	60268-1-Ig
F4/80	Rabbit	IF, IHC	abcam	ab300421
CD86	Rabbit	IF	abcam	ab239075
CD206	Rabbit	IF	abcam	ab64693
MPO	Rabbit	IHC	abcam	ab208670
LY6G	Rabbit	IHC	abcam	ab238132
Flag	Rabbit	WB	ABclonal	AE095
HA	Rabbit	WB	ABclonal	AE105

Supplemental Table S4. Primer Sequences of qPCR Used in This Study

Gene	Species	Forward primer (5′→3′)	Reverse primer (5′→3′)
Actb	Mouse	GGCTGTATTCCCCTCCATCG	CCAGTTGGTAACAATGCCATGT
F13Aa1	Mouse	AGATACTCCAGCAAGCACCT	CCTCAGTTGGGAGGTCCACT
Nlrp3	Mouse	CTACGGCCGTCTACGTCTTC	GGCCAAAGAGGAATCGGACA
Il1b	Mouse	GCAACTGTTCCTGAACTCAACT	ATCTTTTGGGGTCCGTCAACT
Nos2	Mouse	GTTCTCAGCCCAACAATACAAGA	GTGGACGGGTCGATGTCAC
Cxcl10	Mouse	CCAAGTGCTGCCGTCATTTTC	GGCTCGCAGGGATGATTTCAA
Tnf	Mouse	CTGGATGTCAATCAACAATGGGA	ACTAGGGTGTGAGTGTTTTCTGT
Il6	Mouse	TGGGGCTCTTCAAAAGCTCC	AGGAACTATCACCGGATCTTCAA
Sphk1	Mouse	ATGGAACCAGTAGAATGCCCT	TCCGTTCGGTGAGTATCAGTTTA
Sphk2	Mouse	CACGGCGAGTTTGGTTCCTA	CTTCTGGCTTTGGGCGTAGT
Sptlc1	Mouse	ACGAGGCTCCAGCATACCAT	TCAGAACGCTCCTGCAACTTG
Sptlc2	Mouse	AACGGGGAAGTGAGGAACG	CAGCATGGGTGTTTCTTCAAAAG
Asah1	Mouse	CGTGGACAGAAGATTGCAGAA	TGGTGCCTTTTGAGCCAATAAT
Asah2	Mouse	GCAAAGCGAACCTTCTCCAC	ACTGGTAACAAACAAGAGGGTGA

Supplemental Table S5. Primer Sequences of ChIP-PCR Used in This Study

Gene	Species	Forward primer (5′→3′)	Reverse primer (5′→3′)
Il1b	Mouse	CACAGAAGCACCATCCAGT	AGATGCACACCCAGAAGTG
Hif1a	Mouse	CTGGGCAAACTGTTACCG	CCTGCAAGAAACGCTGAA
